# Rapid generation and selection of Cas9-engineering TRP53 R172P mice that do not have off-target effects

**DOI:** 10.1186/s12896-019-0573-z

**Published:** 2019-11-08

**Authors:** Guoxing Zheng, Qingqing Zhu, Junchao Dong, Xin Lin, Chengming Zhu

**Affiliations:** 1 The Seventh Affiliated Hospital, Sun Yat-sen University, Shenzhen, 510275 Guangdong China; 2 Tsinghua-Peking Center for Life Sciences, Beijing, 100084 China; 3 Institute for Immunology, Department of Basic Medical Sciences, School of Medicine, Tsinghua University, Beijing, 100084 China

**Keywords:** Human genetic diseases, Mouse model, TRP53-R172P mutation, CRISPR/Cas9 system, Off-target effects

## Abstract

**Background:**

Genetic mutations cause severe human diseases, and suitable animal models to study the regulatory mechanisms involved are required. The CRISPR/Cas9 system is a powerful, highly efficient and easily manipulated tool for genetic modifications. However, utilization of CRISPR/Cas9 to introduce point mutations and the exclusion of off-target effects in mice remain challenging. TP53-R175 is one of the most frequently mutated sites in human cancers, and it plays crucial roles in human diseases, including cancers and diabetes.

**Results:**

Here, we generated TRP53-R172P mutant mice (C57BL/6 J, corresponding to TP53-R175P in humans) using a single microinjection of the CRISPR/Cas9 system. The optimal parameters comprised gRNA selection, donor designation (silent mutations within gRNA region), the concentration of CRISPR components and the cellular sites of injection. TRP53-R172P conversion was genetically and functionally confirmed. Combination of TA cloning and Sanger sequencing helped identify the correctly targeted mice as well as the off-target effects in the engineered mice, which provide us a strategy to select the on-target mice without off-target effects quickly and efficiently.

**Conclusions:**

A single injection of the this optimized CRISPR/Cas9 system can be applied to introduce particular mutations in the genome of mice without off-target effects to model various human diseases.

## Background

Humans suffer from thousands of genetic disorders, which arises from various mutations in the genome. Among them, single-gene mutations account for over 6000 human monogenic disorders according to Online Mendelian Inheritance in Man (OMIM, https://www.omim.org/). Therefore, suitable animal models are urgently needed to elucidate the regulatory mechanisms of genetic mutations in the development and progression of human diseases.

### p53 mutations in cancers

Cancers that involve abnormal cell growth and have the potential to spread throughout the body, affected 90.5 million people and caused 8.8 million deaths [1, 2] in 2015. Oncogenes and tumour suppressor genes are the two major groups of genes contributing to the transformation of normal cells into malignant cells. The *Tp53* tumour suppressor gene is the most frequently mutated gene in cancers [3, 4], highlighting its importance in cancer generation. Normally, TP53 is sequestered by the negative regulator, mouse double minute 2 homolog (MDM2). Oncogenic events, such as DNA damage, or other stresses release the TP53 protein from the MDM2 complex. Subsequently, TP53 induces cell cycle arrest, initiates DNA damage repair processes to fix the damage or eliminates irreparable cells through senescence or apoptosis [5]. Activated TP53 transactivates the expression of the downstream gene *p21*, which directly binds to cyclin/cyclin-dependent kinase (CDK) complexes and inhibits their kinase activity, thereby leading to cell cycle arrest at the G1/S transition checkpoint [6]. While a variety of mutations of *Tp53* have been found to contribute to malignant progression, the most common ones are all single nucleotide missense mutations which are corresponding to the DNA-binding region of TP53 [7]. Among the 6 hotspot amino acids, Arg175Pro (R175P) substitution leads to completely defective initiation of apoptosis but partially retains the function of cell cycle arrest [8, 9]. The mouse model of this TP53 mutant escapes the early onset of spontaneous tumorigenesis [10] but develops diabetes [11] as well as colon adenocarcinomas [12] upon the deficiency of nonhomologous end-joining (NHEJ). These findings suggest that the mouse model of the human TP53 R175P mutant is valuable to explore the influences of TP53’s ability of cell cycle arrest in human diseases, including cancer and diabetes.

### Genetic editing via CRISPR/Cas9

The Clustered Regularly Interspaced Short Palindromic Repeats (CRISPR) has been demonstrated to be a powerful tool in genomic editing since its first application in human cells [13, 14]. CRISPR system is the prokaryotic immune system and has been identified in 40% of sequenced bacteria and 90% of sequenced archaea [15]. The CRISPR associated protein 9 (Cas9) endonuclease is a simple CRISPR system from *Streptococcus pyogenes* that contains four components, and it was re-engineered into an even simpler two-component system [16]. The efforts of geneticists have shown that the Cas9 system is highly efficient, easily manipulated, and flexible. This system has been widely used in genomic editing of various organisms, including mice [17]. The genome-editing system includes Cas9, gRNA, and a donor (an optional section of DNA repair template). The gRNA of 20 nucleotides can be designed to target the genomic sites of interest with a 5′-NGG-3’protospacer adjacent motif (PAM) sequence [16]. The gRNA directs Cas9 to target sites where it cleaves the DNA and produces double strand breaks (DSBs). The DNA damage is repaired by NHEJ which causes insertions or deletions randomly, or by homology-directed repair (HDR) with a donor to generate the desired mutations in the genome [14]. While the engineering of the mice via CRISPR/Cas9 system has been widely adopted and reported, the functional confirmation of genetic mutations as well as the identification and excluding of the off-target effects in descendant generations remain unexplored. In this study, a new insight of the application of CRISPR/Cas9 system was provided for murine genetic modification which minimizes the off-target effect and can be recommended to future application of modelling human diseases.

### Objectives

This study was to explore the practicability of constructing genetically modified mice of TRP53-R172P by a single injection of the CRISPR/Cas9 system. The single amino acid substitution would be validated genetically and functionally, and the excluding of the off-target effect in the descendant generation would be confirmed.

## Results

### Generation of TRP53R172P mice with a single microinjection of CRISPR/Cas9 system

**Fig. 1:**
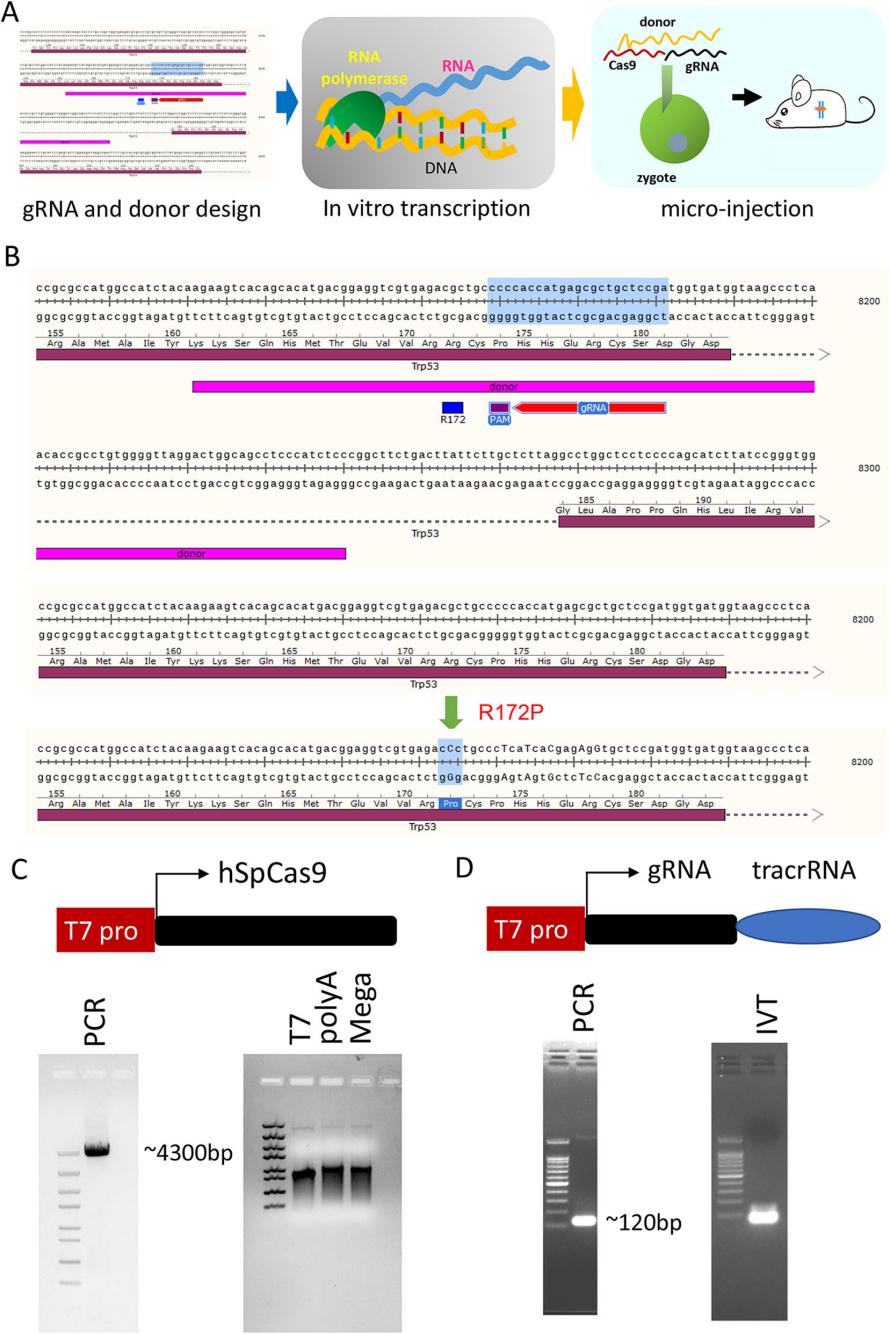
Introduction of the R172P substitution in TRP53 locus via a single injection of the CRISPR/Cas9 system. **a** Procedure of construction of KI mice: Step 1) Design gRNA and donor for mouse engineering; Step 2) In vitro transcription to generate mRNA of *Cas9* and gRNA; Step 3) Prepare zygotes for microinjection and inject *Cas9* mRNA, gRNA and donor into zygotes as well as transplant obtained zygotes into foster mother mice. **b** A schematic illustration shows the designation of gRNA and donor in TRP53 R172P KI mouse engineering. The gRNA closest to the mutation site was selected where the mutation site(g- > C) locates5 nucleotides downstream of gRNA NGG (PAM site). A 120-nucleotide length oligo donor was used, which covered the gRNA and mutation site (top panel). The donor carried 6 silent mutations, which do not cause amino acid replacement, in the gRNA region and a G- > C mutation to generate the R172P substitution of the TRP53 tumour suppressor (bottom panel). **c** The PCR product contained a T7 promoter before the *hSpCas9* cDNA with an approximate length of 4300 bp (top panel). PCR amplification of *hSpCas9* cDNA from the pX260 vector was performed using the Phusion high-fidelity PCR kit, and the single band product with the correct length is shown in the agarose gel (left panel). The agarose gel image (right panel) shows that the MEGAclear kit purification and poly A addition products had the same length and were longer than the in vitro transcription product of T7 RNA polymerase. **d** The PCR product contained a T7 promoter, gRNA and tracrRNA with an approximate length of 120 bp (top panel). PCR amplification of gRNA DNA with high-fidelity PCR kit resulted in a single band product with the correct length as shown in the agarose gel (left panel). The in vitro transcription product of gRNA was validated by agarose gel electrophoresis (right panel)

To generate mice with a desired mutation using the CRISPR/Cas9 system, the selected gRNA and Cas9 were in vitro transcribed (IVT) into mRNAs, which were then microinjected into fertilized eggs along with single-strand oligonucleotide donor (SSOD) (Fig. 1a). In the mouse genome of *Trp53*, a single nucleotide mutation of 515G- > C results in a R172P transition (corresponding to TP53-R175P in humans). The closest gRNA of the mutation site, locating 5 nucleotides upstream was chosen and a 120-nucleotide donor carrying the 515G- > C mutation (Fig. 1b, top panel) was designed. To eliminate the re-targeting by gRNA, 4 nucleotides in the gRNA sequence and one nucleotide in PAM were silently mutated in the donor (Fig. 1b, bottom panel).

Humanized *Cas9* was PCR amplified and in vitro transcribed into mRNA (Fig. 1c, left panel), *Trp53* gRNA together with tracrRNA was PCR amplified and in vitro transcribed into mRNA (Fig. 1c, right panel). *Cas9* mRNA (40 ng/μl), sgRNA (17.5 ng/μl), and donor oligos (60 ng/μl) were mixed and directly micro-injected into both nuclei and cytoplasm of mouse zygotes at one time. Two hundred ten fertilized eggs were injected, among which 167 zygotes grew into 2-cell stage embryos (79.5% survive), and 26 pups were finally born (16.7%) (Table 1, top line).

**Table 1:** Single targeting of TRP53-R172P mice with CRISPR/Cas9 system

Cas9/gRNA/donor (ng/μl)	Survival/ Injected zygotes	Transferred embryos/ Recipients	Newborns
40/17.5/60	167/210	156/6	26
Total mice	WT	KO (double peaks)	KI
26	15/26	11/26	3/26

### Identification of the designated TRP53 R172P mice

**Fig. 2:**
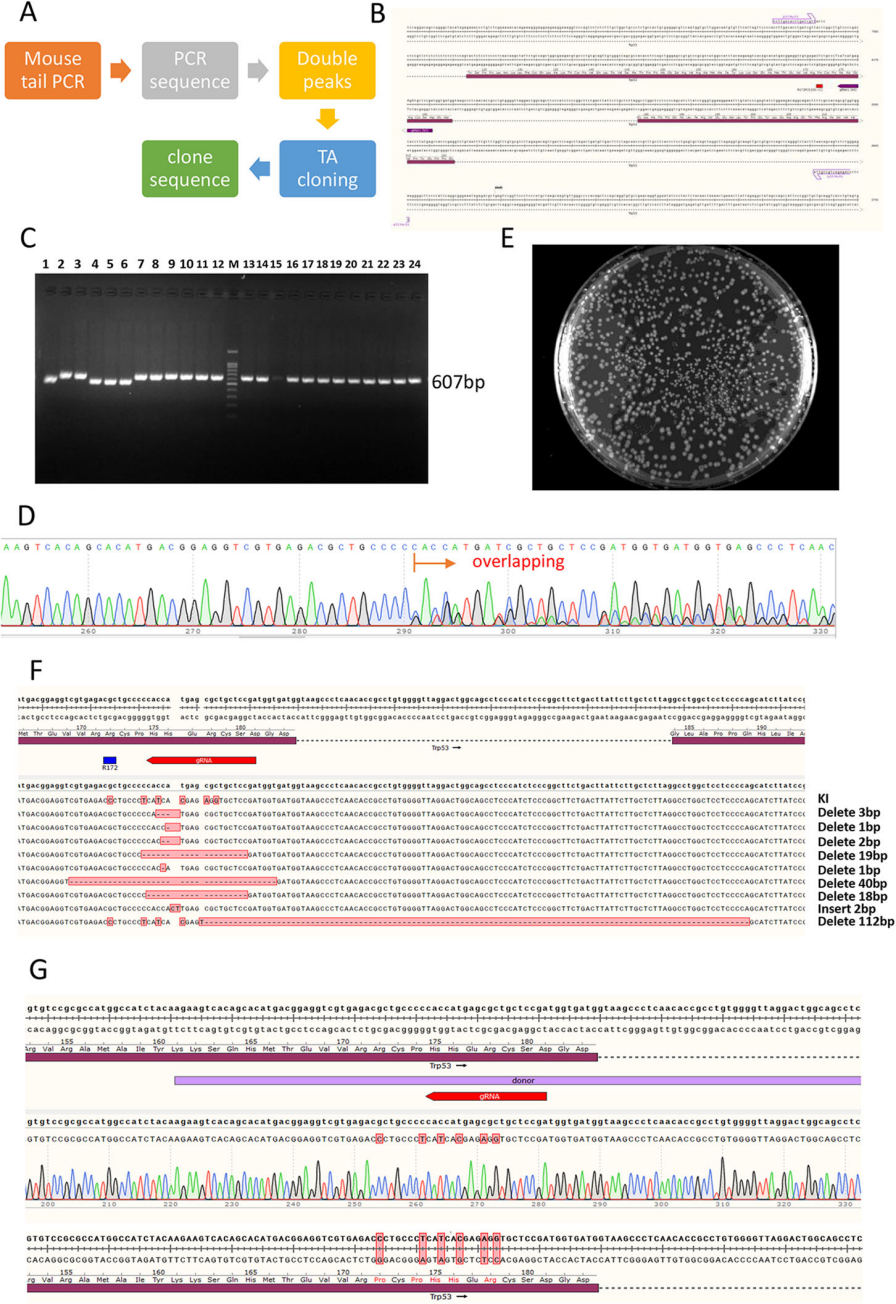
Identification of positive targeting mice for the CRISPR/Cas9 genomic engineering. **a** If DNA sequencing of mouse tail PCR products produced double peaks (frame shift in one allele), the products were subjected to TA cloning, and the further sequencing of independent clones would provide the detailed genomic information of the mosaic mice. **b** The R172P mutation and gRNA site were framed with a primer pair (607 bp) in the genome for PCR identification. **c** The electropherogram (bottom panel) shows the PCR identification of Cas9 engineered mice. **d** The direct sequencing result of PCR products shows the continuously overlapping peaks (double peaks) caused by different allelic substitutions. **e** The LB agar plate shows the *E. coli* bacterial clones during TA cloning. **f** DNA sequencing of TA clones identified 10 different genomic mutations after Cas9targeting. **g** DNA sequencing of TA clones confirmed that the donor carried 6 synonymous mutations in the gRNA region and a G- > C mutation in the mouse genome, which produced the R172P mutation in the TRP53 of tumour suppressor

To identify the targeted mice, the PCR products of pups’ tails showing continuously overlapping peaks (so called double peaks) in Sanger sequencing were subjected to TA cloning and further sequenced (Fig. 2a). One primer set framing the mutation site (Fig. 2b) was utilized to amplify the genome of mouse tails (Fig. 2c). Sanger sequencing of PCR products of 11 mice had double peaks (Fig. 2d). These products were subjected to TA cloning (Fig. 2e) and further analysed. Ten different genomic mutations (Fig. 2f), including knock-in (KI), insertion, and 8 deletions of different lengths were found in the founder mice. Among these mice, 3 (Table 1, bottom line) had the genomic recombination of donor carrying the G515C (pR172P) mutation along with 5 synonymous mutations in gRNA region (Fig. 2g).

### Confirmation of the TRP53 R172P substitution in various tissues and descendants

**Fig. 3:**
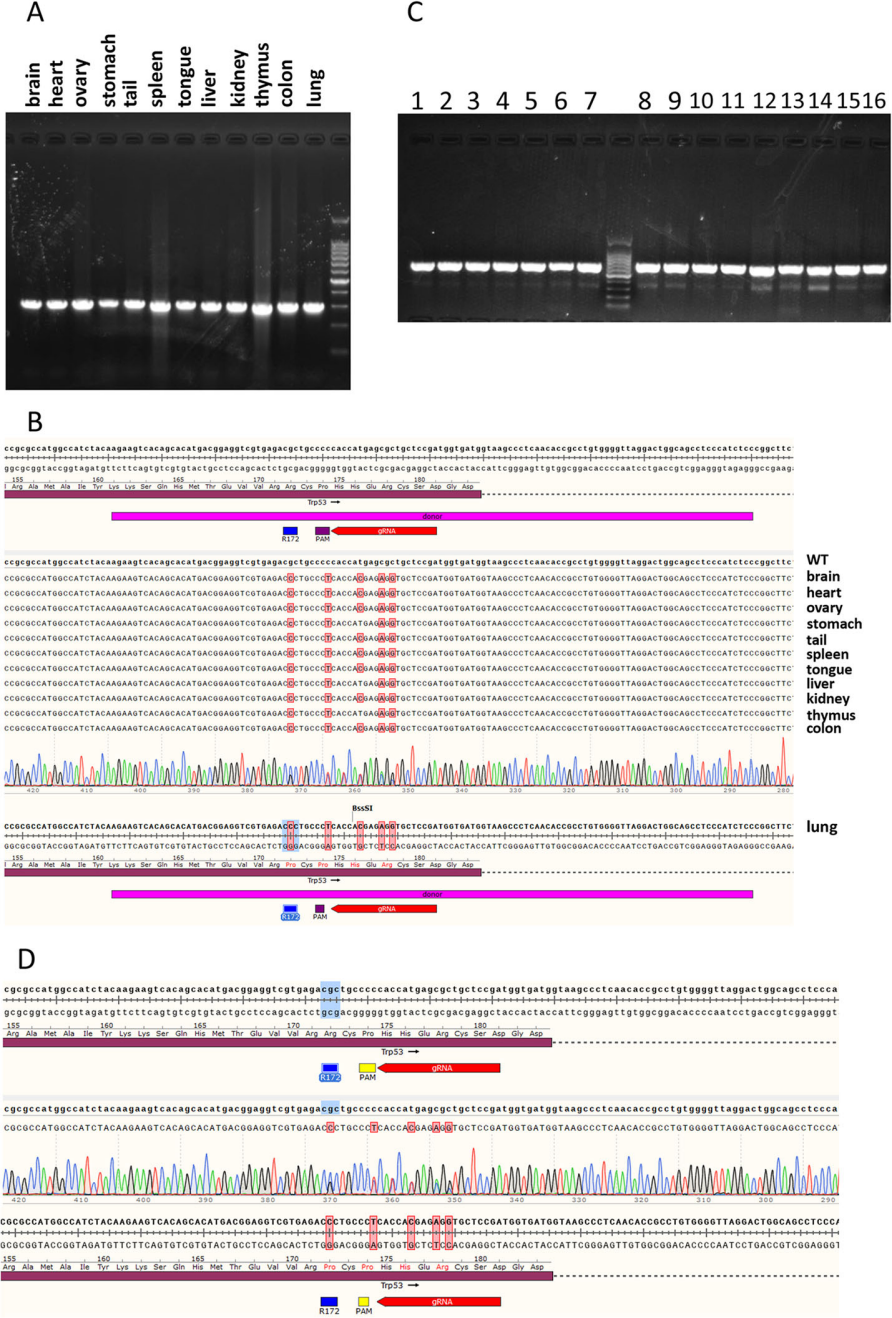
Validation of the TRP53 R172P KI in various tissues of mice and in the mice of Generation 1. **a** The electropherogram shows the PCR identification of various tissues of KI mice with the product of 607 bp. **b** Sanger DNA sequencing of PCR products confirmed the desired mutations in the different tissues including brain, heart, ovary, stomach, tail, spleen, tongue, liver, kidney, thymus, colon and lung. **c** The agarose gel image shows the PCR identification of Generation 1 offspring of Cas9 engineered mice with the product of 607 bp. **d** DNA sequencing of PCR products confirmed the expected mutations in the genome of G1 mice

According to the sequencing results of the TA clones, these KI mice were all mosaic that also carried various mutations in their genome. The KI mice were mated with wild-type mice to isolate the correct targeting allele in their offspring. To validate the TRP53 R172P mutation in the whole body of generation 1 mice, genomes from various tissues including brain, heart, ovary, stomach, tail, spleen, tongue, liver, kidney, thymus, colon and lung were PCR amplified (Fig. 3) and sequenced. The results showed that all these different tissues contain the same defined mutations in the genomes (Fig. 3b). In addition, the TRP53 R172P substitution in descendants of F1 KI mice was also confirmed (Fig. 3c and d). These results suggest that the TRP53-R172P mutant mice were achieved.

### Examination of the off-target effects of targeting mice

**Fig. 4:**
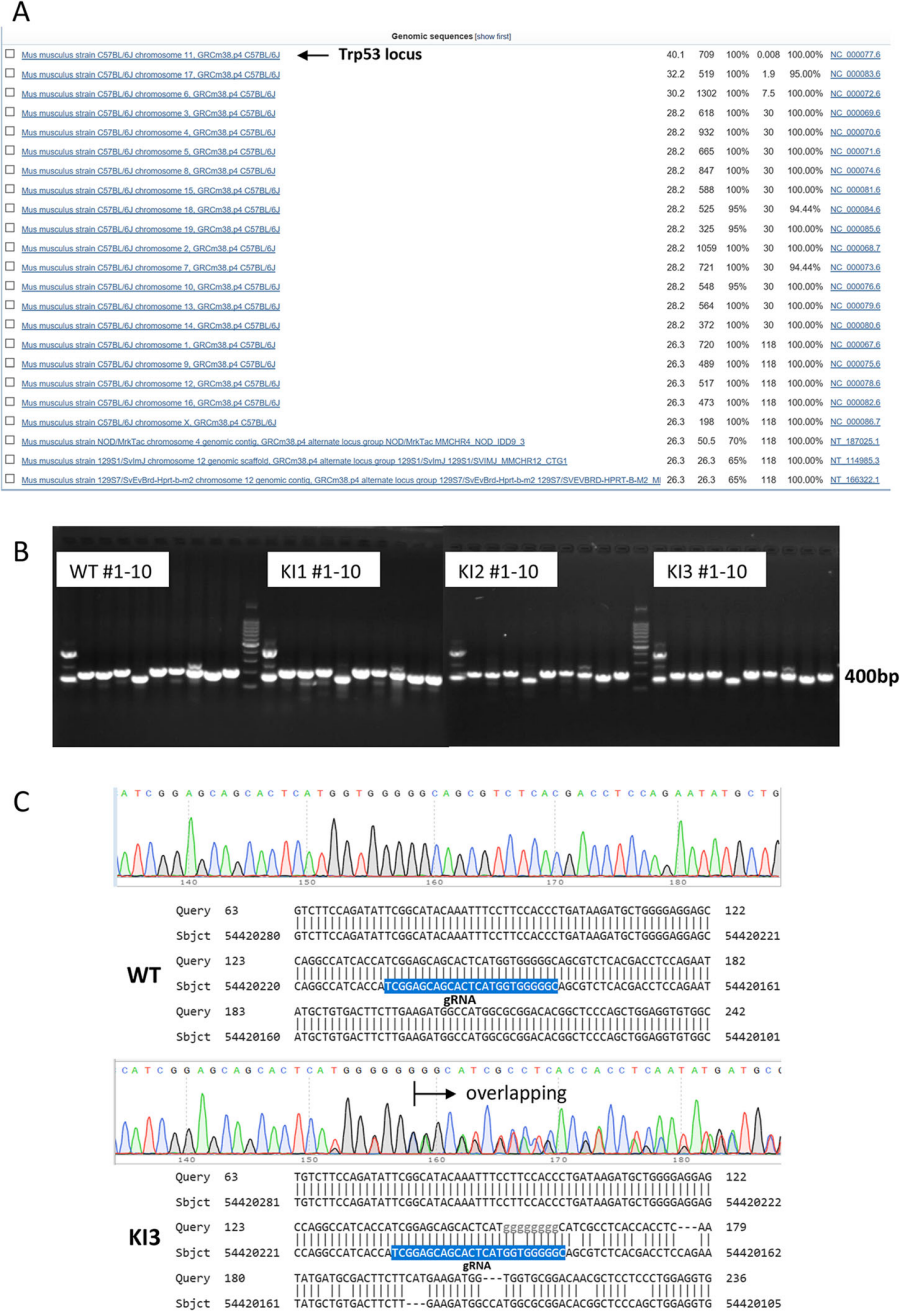
Analysis of the potential off-target effects in the genome of TRP53 R172P KI mice with PCR and Sanger sequencing. **a** BLAST of gRNA applied in the study with mouse genome showed 23 matching loci with the *Trp53* locus on the top. **b** The top 10 putative off-target loci in genome were PCR amplified with their own specific primer pairs and separated with electrophoresis on agarose gel. **c** Sanger Sequencing of the target PCR products (short band) of #1 off-target locus (Trp53 pseudogene) showed overlapping peaks in KI1 and KI3 mice but not in WT and KI2 mice. BLAST of the PCR products of #1 off-target locus (Trp53 pseudogene) showed tons of mismatches since the gRNA region in KI1 and KI3 mice but not in WT and KI2 mice, indicating the off-target effects of locus #1 in KI1 and KI3 mice. The results of KI2 and KI3 are shown on Additional file 1: Figure S1

To explore the potential off-target loci, the gRNA used for the targeting was aligned with mouse genome by BLAST (Basic Local Alignment Search Tool). Twenty-three loci are found to match with gRNA, among them the top 1 is *Trp53* locus (Fig. 4a). The specific primer pairs were designed to amplify the top 10 loci except *Trp53*. PCR amplification of WT and 3 KI mice with the 10 specific pairs of primers was performed (Fig. 4b). The PCR products were subsequently subjected to Sanger sequencing and aligned with mouse genome to determine the off-target effects of all the 10 loci. No off-target effects of #2–10 loci were observed on all the 4 mice (Additional file 5: Table S1). While no off-target effects of #1 locus were found in WT and KI2 mice, there were double peaks in the sequencing of KI1 and KI3 mice (Fig. 4c, Additional file 1: Figure S1). BLAST the DNA sequences of KI1 and KI3 mice with mouse genome showed lots of mismatches after gRNA region (Fig. 4c, Additional file 1: Figure S1), indicating the frame shift of one allele near gRNA. The result suggests that there are off-target effects of #1 locus in KI1 and KI3 mice but not in WT and KI2 mice.

### Analysis of the off-target effects of descendants arising from different parents

**Fig. 5:**
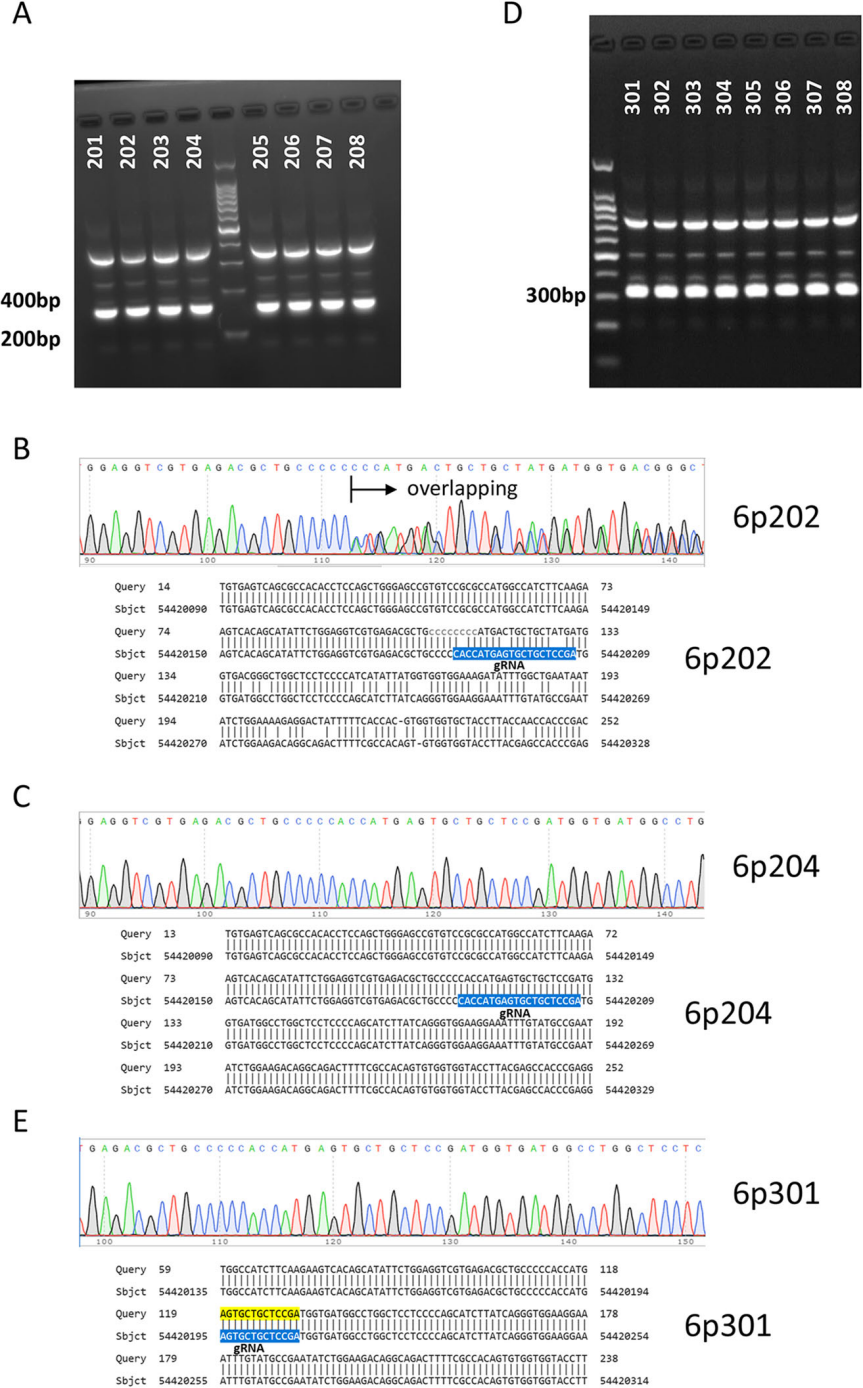
Analysis of the off-target effects in the descendants of mice with or without off-target effects. **a** The #1 off-target locus (Trp53 pseudogene) in 8 descendants of mice with off-target effect (mouse# KI3) was PCR amplified with the specific primer pair and separated with electrophoresis on agarose gel. **b** Sanger Sequencing and BLAST showed the representative off-target effect detected in one offspring (mouse #6p202). **c** Sanger Sequencing and BLAST showed the representative on-target effect detected in one offspring (mouse #6p204). **d** The #1 off-target locus (Trp53 pseudogene) in 8 descendants of mice with on-target effect (mouse #6p204) was PCR amplified and assayed on agarose gel. **e** Sanger Sequencing and BLAST showed the representative on-target effect (mouse #6p301) detected in all 8 descendants of mice with on-target effect (mouse #6p204)

**Fig. 6:**
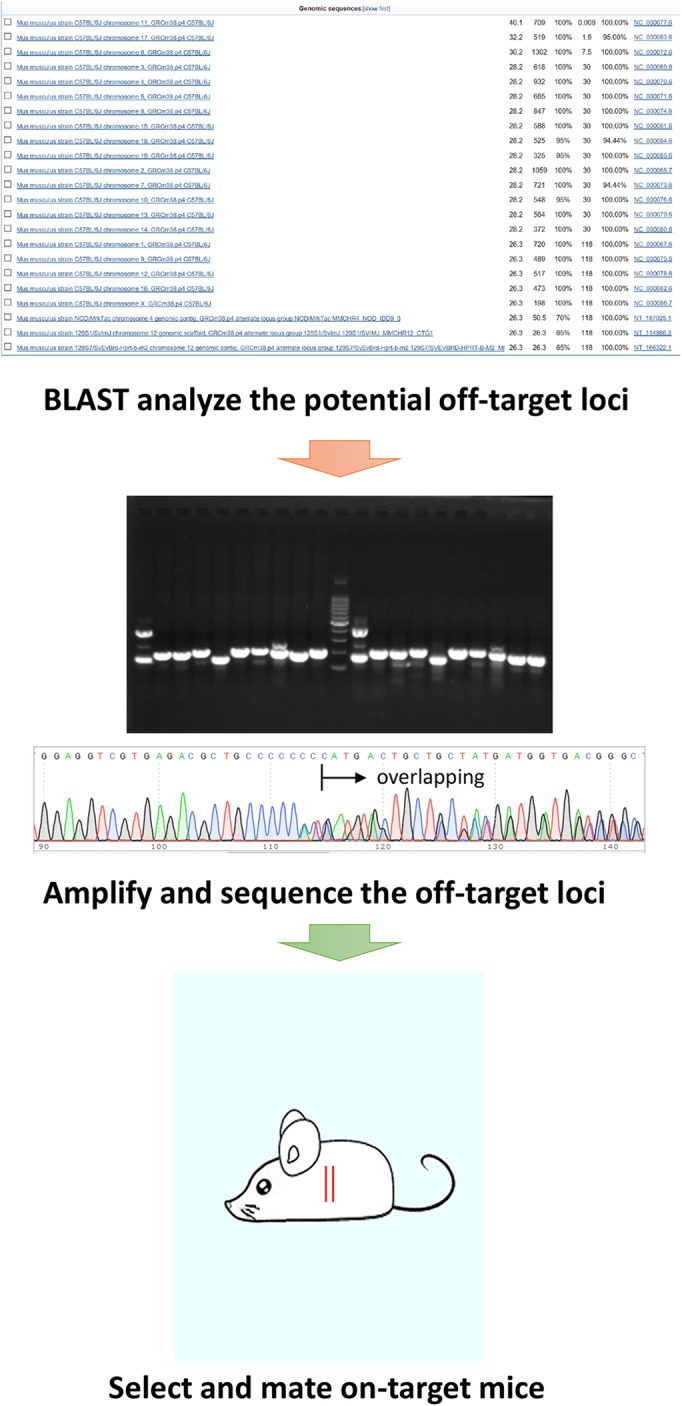
The strategy to exclude the off-target effects in the descendants of the engineering mice based on the CRISPR/Cas9 system. To avoid the off-target effects in the CRISPR mice, the following strategy is performed. Step 1) BLAST search the potential matching loci in mouse genome with the gRNA applied for mouse engineering; Step 2) Amplify and sequence the potential off-target loci with specific primers in generation 1 mice; Step 3) Select those mice that do have off-target effects to be further studied. The strategy can be applied on any mouse constructed via the CRISPR/Cas9 system including Non-homologous end joining, NHEJ-drive knockout (frame shift mutation) and Homology directed repair, HDR-drive knock-in (donor replacement) mice

In order to examine whether the off-target effects can be inherited by the descendants, we detect the off-target effects on the offspring of KI3 mouse. PCR amplification of #1 locus was performed on 8 KI descendants of KI3 mouse (Fig. 5a). Sanger sequencing and BLAST alignments showed that 4 of 8 mice had off-target effects (Fig. 5b and c, Additional file 1: Figure S1), suggesting that the off-target effects on KI3 mouse is on one allele and can be delivered to its offspring. Aiming to exclude the off-target effects in the descendants of mice generated via the CRISPR/Cas9 system, we asked whether the offspring of mouse without off-target effects are free of off-target effects. The 8 descendants (4 WT, 4 KI) of 6p204 mouse without off-target effects were analysed. PCR amplification of #1 locus (Fig. 5d), Sanger sequencing as well as BLAST alignments (Fig. 5e) were performed. As we expected, all the 8 descendants did not have the off-target effects of #1 locus (Additional file 2: Figure S2). This provide us one strategy to exclude the off-target effects in generation 1–2 descendant mice derived from the CRISPR/Cas9 system. The strategy is to identify of the potential off-target effects in generation 1 mice and select the mice without off-target effects for further study (Fig. 6). The strategy will exclude the off-target effects in a short term and can be applied to other engineering mice based on the CRISPR/Cas9 system.

### Validation of UV radiation hypersensitivity of TRP53 R172P mutant

**Fig. 7:**
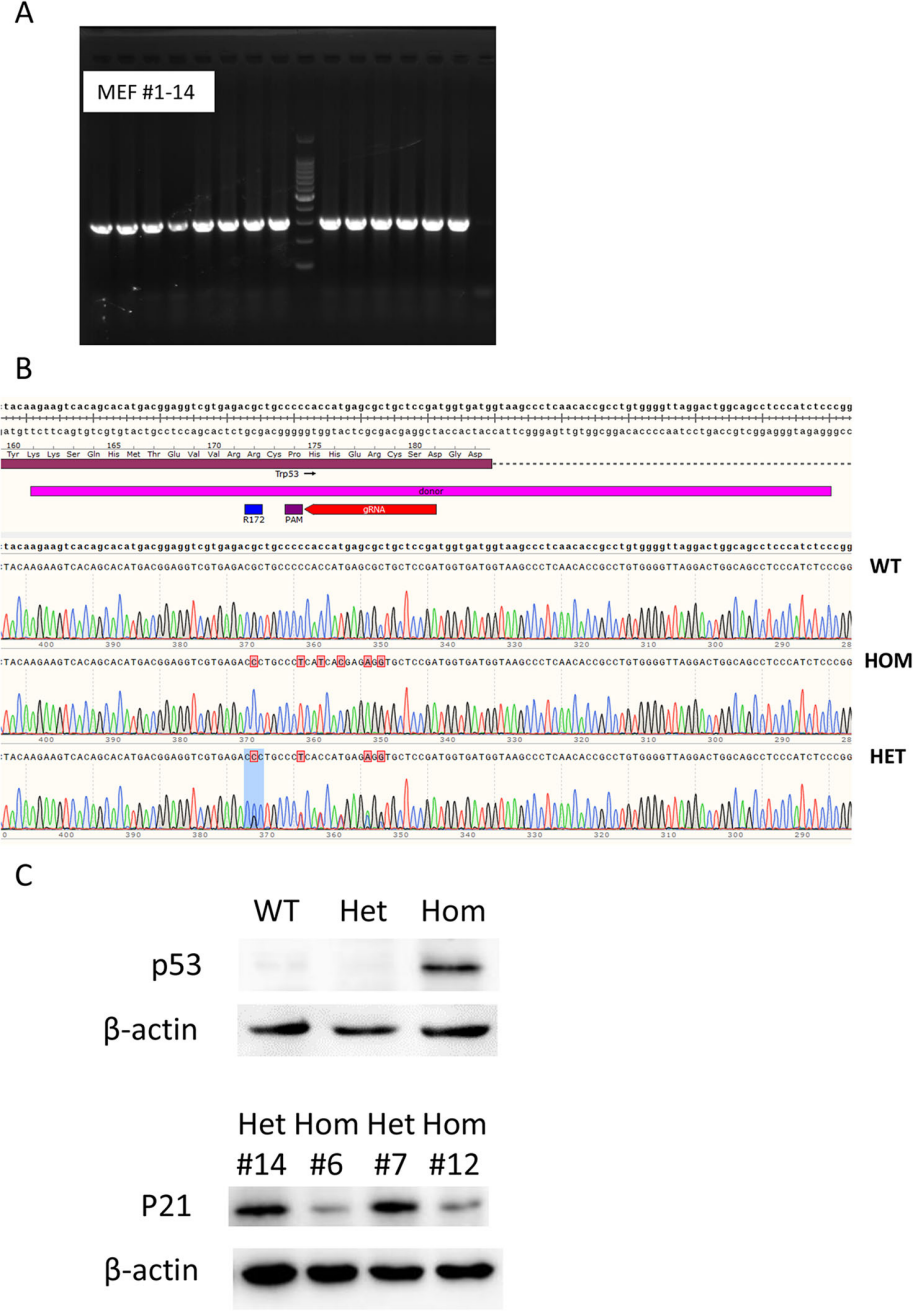
Functional confirmation of the UV radiation hypersensitivity of TRP53 R172P. **a** The agarose gel image shows the PCR identification of mouse embryonic fibroblast (MEF) cells from 14 E13.5 embryos from the mating via TRP53 R172P heterozygous male and female mice. **b** Sanger DNA sequencing of PCR products identified the wild type (WT), TRP53 R172P heterozygous (HET) and homozygous (HOM) MEFs. **c** Western blotting shows the expression level of p53 and p21 in WT (#1), HET (#9) and HOM (#8) MEFs, suggesting the UV radiation hypersensitivity of TRP53 R172P HOM

p53^R172P^ mouse embryonic fibroblasts (MEFs) are hypersensitive to ultraviolet (UV) radiations, indicated by significantly higher induction of p53 [18]. To confirm the responses of TRP53 R172P mutant to UV exposure in our KI mice, we treated MEF cells arising from the KI mice with UV light and detected the p53 protein level. Totally 14 E13.5 embryos were found in the uterus of TRP53 R172P heterozygous mother mated with TRP53 R172P heterozygous male. The genomic DNA of embryonic heads was PCR amplified (Fig. 7a). Sanger sequencing of the products indicated three genotypes of MEFs -wild type (WT), homozygous (HOM), heterozygous (HET) (Fig. 7b). The MEFs were exposed to UV lights for 15 s and cultured for 24 h before lysis. Immunoblotting of p53 showed that compared to both WT and HET MEFs, the p53 level significantly increased upon UV radiation (Fig. 7c, Additional file 4: Figure S4) in HOM cells, in line with previous observations. Also, the expression of another indicator p21, which is the downstream effector of p53 during cell apoptosis, was significantly decreased in HOM MEFs upon UV radiation, consistent to previous results. The result suggests that the KI mice we obtained have the function of UV radiation hypersensitivity.

## Discussion

The present study generated tumour suppressor gene TRP53 R172P mutant mice by a single injection of CRISPR-cas9 system. The present results suggested that microinjection of 200 zygotes is sufficient to produce knock-in mice with genetic point mutations. The efforts to obtain this mouse line included optimization of the designation of gRNA and donor as well as confirmation of the results step by step. It has demonstrated that the procedure worked well and can be applied to generate mouse models for other human genetic diseases.

### gRNA and donor designation

The selected gRNA has great influence on the targeting efficiency of CRISPR-Cas9 in mice. To generate a point mutation in the genome, mutation sites must be close enough to gRNA. Mutation sites closer to the Cas9 cleavage site (nucleotide4 before PAM) will have higher genomic knock-in efficiency. Donor designation was also optimized through introduction of synonymous mutations in the gRNA region, which abolished the effect of secondary targeting of Cas9 on successfully knock-in genomic sites. To ensure escape from Cas9 targeting, the donor comprised one mutation at PAM and mutations of at least 3 nucleotides in the gRNA in this study.

### Injection concentration and cellular sites

For genomic knock-in, the mixture of RNA and DNA needs to be injected into both the nuclei, where the homologous directed recombination (HDR) of donor occurs, and cytoplasm, where the Cas9 mRNA is translated into enzyme, of fertilized eggs. A balance of the concentrations of injection components also exists. Higher concentrations produce higher targeting efficiency but lead to higher percentage of zygote death. The concentration used in the present study was optimized to result in good targeting efficiency but also to generate a sufficient number of surviving mice.

### Elimination of the off-target effects

Several publications have reported that most mice generated from direct introduction of Cas9 mRNA and sgRNA into zygotes are genetic mosaics, that is, one mutant mouse is composed of cells carrying different sets of mutations [19–21]. Similarly, the genotypic mosaicism was found in founder mice derived from injected zygotes in the present study. To identify 1st generation KI mice, TA cloning technology, which can effectively and efficiently dissect the detailed genomic information of mosaic mice, was applied [22, 23]. DNA sequencing of 6–8 clones offered 3–4 different genotypes in the mosaic mice. One common criticism of CRISPR/Cas9 system is the off-target effects. Same as the genetic modification, the off-target effects of CRISPR/Cas9 system can be traced, identified (Additional file 3: Figure S3) and passed on to offspring mice. To exclude the off-target effects of the CRISPR/Cas9 system, we develop a new rapid approach (Fig. 6): screen the potential off-target loci in generation 1 mice and select those that do not carry off-target effects for further applications. This approach resolves the off-target problems in a short time and at an efficient manner, suggesting its wide utilizations in future for engineering mice based on CRISPR/Cas9 system.

### The advantages of Cas9 over conventional methods

Before the discovery of Cas9 editing system, homologous recombination is commonly carried out to introduce the inherit mutations into genome. The procedure comprises several complicated steps, which makes it time consuming and high cost. Comparably, it is straight forward and easy forCas9 mediated genetic editing that does not need the cloning of large genomic fragments. And the identification of correct targeting in Cas9 editing with PCR and Sanger sequencing is much easier than that in homologous recombination with drug selection and Southern blotting. More importantly, the targeting efficiency is significantly higher with Cas9 system and no exogenous elements will be introduced into the genome. Also, the potential off-target effects of Cas9 should be taken into account but can be excluded.

## Conclusions

The Cas9 system is a powerful tool to generate mice carrying genetic mutations for studying the pathology of cancers and other human genetic diseases. To overcome the difficulty of generating KI mice, the optimizations of the donor designation, cellular injection sites and the injection concentration are required to increase the efficiency of successfully targeting. More importantly, we developed an approach to determine and avoid the off-target effects in Cas9 engineered mice in a short term and an efficient manner. The approach can be applied in any engineered mouse derived from Cas9 targeting, no matter for gene knockout or donor substitution. In future, the benefit of Cas9-mediated production of KI alleles needs to be carefully and systematically evaluated. And It is likely that the Cas9 mouse will have more applications beyond cancer field.

## Methods

### In vitro transcription of Cas9 and gRNA

The *Cas9-*coding region was PCR amplified with Phusion DNA polymerase from the pX260 plasmid (Addgene) using the Cas9-F primer containing the T7 promoter and Cas9-R primer (Additional file 6: Data 1). *Cas9* PCR products were purified with phenol-chloroform. Following the manufacture’s manual, in vitro transcription (IVT) of *Cas9* was performed using the mMESSAGE mMACHINE T7 Ultra Kit (Ambion, AM1345). Agar gel electrophoresis and nanodrop analysis were used to verify the quality and concentration of the obtained mRNA purified with MEGAclear kit (Ambion, AM1908). Purified PCR products of T7-gRNA for *Trp53* were used as a template for IVT using the MEGAshortscript T7 kit (Ambion, AM1354). gRNA was purified with the MEGAclear kit (Ambion, AM1908) and resuspended in endonuclease-free water. Agarose gel electrophoresis and nanodrop analysis were used to verify the quality and concentration.

### Source of animals

The female and male C57BL/6 mice, ICR mice used in this study were housed and bred in the Animal Center of Tsinghua University.

### Zygote injection of Cas9 the system

C57BL/6 J female mice and ICR mouse strains were used as embryo donors and foster mothers, respectively. Superovulated 8-week-old female C57BL/6 J mice were mated to C57BL/6 J males, and the fertilized eggs from oviducts were collected. *Cas9* mRNA (40 ng/μl), *Trp53* gRNA (17.5 ng/μl), and donor oligos (60 ng/μl) were mixed in 20 μl of nuclease-free H_2_O and centrifuged at 12000 rpm for 2 min. The supernatant was transferred into a new Eppendorf tube and microinjected into both the nuclei and cytoplasm of zygotes at the pronuclei stage in M2 medium (Sigma).210 Injected zygotes were cultured in KSOM medium at 37 °C and 5% CO_2_ for 1 day. The surviving embryos at the 2-cell stage were transferred into uterus of 6 pseudopregnant female mice. Totally, 26 mice were born.

### Euthanasia of the animals

The euthanasia of adult mice was performed via a gradual fill of CO_2_ at a rate of about 20% chamber volume per minute, and maintained for more than 5 min. The death of mice was verified before removing the mice from the CO_2_ chamber.

### TA cloning

PCR products of mouse tail amplification were confirmed on an ethidium bromide-stained agarose gel and subjected to Sanger sequencing. The products with continuous overlapping peaks (so called double peaks) in Sanger sequencing were subjected to TA cloning with pEASY®-Blunt Cloning Kit (Transgene). The ligated products were transformed into Trans1-T1 competent cells which were then plated on LB agar dishes containing ampicillin, and 6–8 bacterial clones from every dish were DNA sequenced for the identification of mouse genomic information.

### Mouse embryo fibroblast (MEF) preparation and UV treatment

Trp53 R172P Heterozygous mouse was mated with the same genotype mouse, the mother was sacrificed at embryo stage E13.5. The embryonic heads were harvested for genome extraction to genotype the MEFs. After remove of limbs and visceral tissues, the embryos were sectioned into small pieces and digested with 0.25%trypsin at 37 °C for 10 min. The trypsinization was stopped by 10% FBS. The cells were isolated by vigorously pipetting and plated at 10 cm dishes before incubation at 37 °C, 5% CO2until 100% confluence. Simultaneously, wild type (WT), Heterozygous (HET) and Homozygous (HOM) MEF cells were exposed to UV light (GE, G36T5L (39 W) UV-C Ultraviolet Germicidal 254 nm LIGHT Bulb Lamp) for 15 s (The time course of UV treatment was optimized before the experiments.). The cells were collected 24 h after UV treatments with nm UV light equipped in the biosafe incubator.

### Immunoblotting

The MEFs were lysed in RIPA buffer containing 50 mmol/L Tris-HCl pH 8.0, 150 mmol/L NaCl, 1%Nonidet P-40, 1% Na-deoxycholale, 0.1% SDS, 1 mmol/L Na3VO4, 1 mmol/L NaF,1 mmol/L PMSF and a protease inhibitor mixture (Roche Diagnostics, Mannheim, Germany). The cell lysates were subjected to separation with SDS PAGE and immunoblotted with specific antibodies of p53 (Cell signalling technology, CST#2524) and β-Actin (Servicebio).

### The raw data collection

All the raw data can be found in the Additional file 7: Data 2.

## Supplementary information

**Additional file 1: Figure S1.** The same off-target effect was found in half of the offspring of mice carrying off-target locus. (A) Sanger Sequencing of the target PCR products (short band) of #1 off-target locus (Trp53 pseudogene) showed overlapping peaks in KI1 mouse but not in KI2 mouse. BLAST of the PCR products of #1 off-target locus (Trp53 pseudogene) showed tons of mismatches since the gRNA region in KI1 mouse but not in KI2 mouse, indicating the off-target effects of locus #1 in KI1 mouse. (B) Sanger Sequencing of the PCR products of #1 off-target locus (Trp53 pseudogene) showed overlapping peaks (indicating off-target effect) in half (4/8) of KI offspring of mice carrying #1 off-target locus. (C) BLAST of the PCR product of #1 off-target locus (Trp53 pseudogene) showed tons of mismatches since the gRNA region (indicating off-target effect) in half (4/8) of KI offspring of mice carrying #1 off-target locus.

**Additional file 2: Figure S2.** No off-target effects were found in all the offspring of mice that did not carry off-target locus. (A) PCR identifications of KI offspring of mice not carrying #1 off-target locus were validated by gel electrophoresis. (B) Sanger Sequencing of the PCR products of #1 off-target locus (Trp53 pseudogene) showed none of overlapping peaks (indicating off-target effect) in all of 8 offspring of mice without off-target effect. (C) BLAST of the PCR product of #1 off-target locus (Trp53 pseudogene) confirmed none of off-target effect in all of 8offspring of mice not carrying #1 off-target locus.

**Additional file 3: Figure S3.** TA cloning and Sanger sequencing dissected the mutations of #1 off-target locus (Trp53 pseudogene). (A) TA clones of PCR products of #1 off-target locus were subjected to Sanger sequencing for analysing the detailed genomic mutations in #1 off-target locus. Sequence alignments showed that there were 75 bp insertion (222–299) in the #1 off-target locus. (B) Sequence alignments showed 3 bp deletion in the #1 off-target locus. (C) Sequence alignments of another clone showed 3 bp deletion in the #1 off-target locus.

**Additional file 4: Figure S4.** p53 level in the MEFs upon the stimulation of UV radiation. The protein levels of p53 in MEFs of various genotypes are compared upon UV stimulation of indicated time. The result showed that the expression of p53 increased in all Homozygous MEF cells. β-Actin worked as normalization control.

**Additional file 5: Table S1.** Summary of the analysis of the potential off-target loci. The top 10 potential off-target loci are PCR amplified and subsequently subjected to Sanger sequencing and aligned with mouse genome. Although no off-target effects of #2–10 loci are found on all the 4 mice, the off-target effects of #1 locus are detected in KI1 and KI3 mice.

**Additional file 6: Data 1.** oligos used in p53 R172P knockin.

**Additional file 7: Data 2.** The raw data collection.

## Data Availability

All data generated or analysed during this study are included in this published article and supplementary information files.

## Abbreviations

Cas9: CRISPR-associated protein 9;
CDK: cyclin-dependent kinase
CRISPR: Clustered Regularly Interspaced Short Palindromic Repeats
DMSO: dimethyl sulfoxide
DSB: double strand break
HDR: homology-directed repair
IVT: in vitro transcription
KI: knock-in
KO: knockout
LB: Luria-Bertani
MDM2: mouse double minute 2 homolog;
NHEJ: nonhomologous end-joining
OMIM: Online Mendelian Inheritance in Man
PAM: protospacer adjacent motif
