# Supplementary figures and images for: Rapid generation and selection of Cas9-engineering TRP53 R172P mice that do not have off-target effects
Source: BMC Biotechnol. 2019 Nov 8;19:74. doi: 10.1186/s12896-019-0573-z (PMC6839086; doi:10.1186/s12896-019-0573-z)

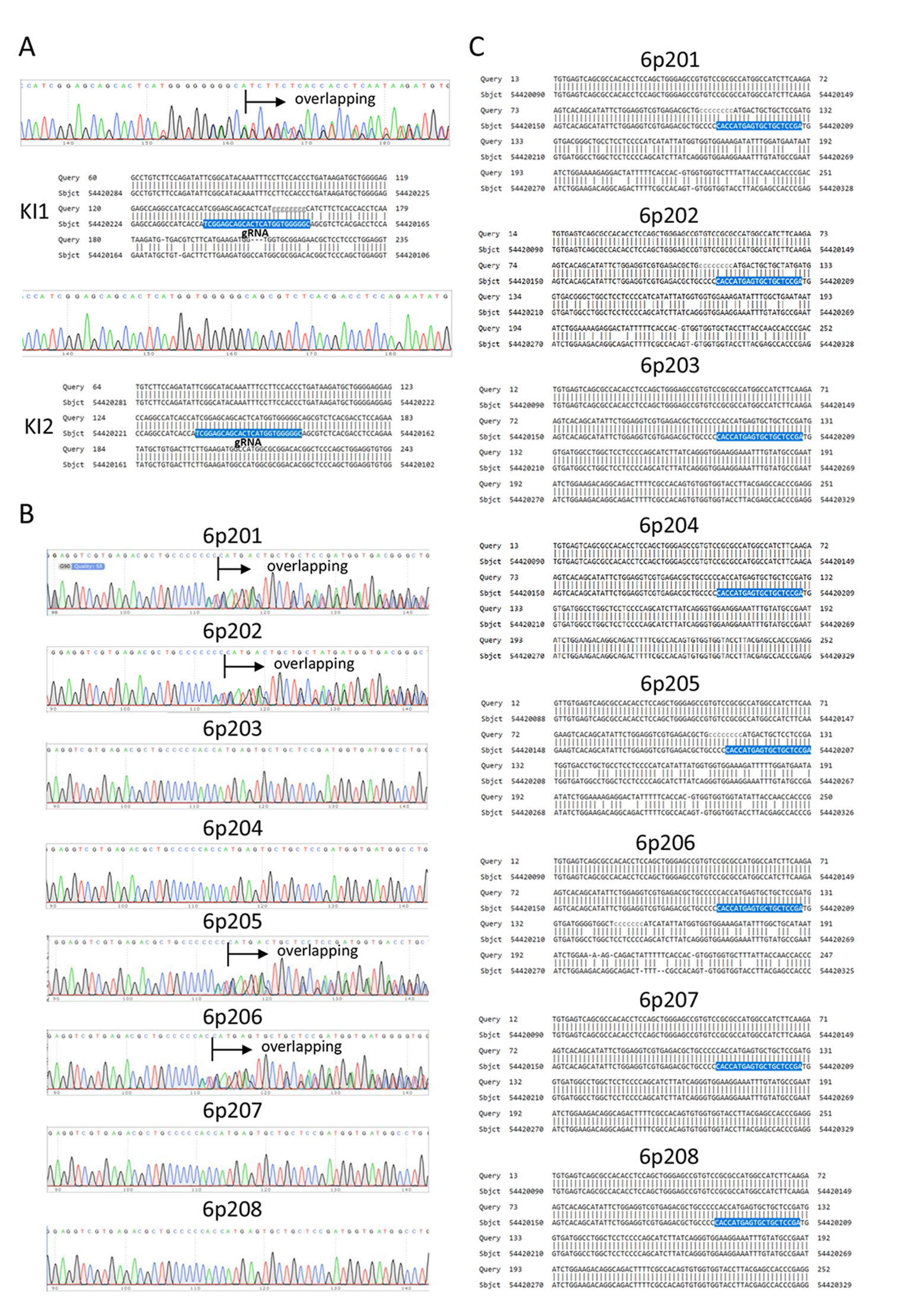

Supplement: Supplementary file 1 — Additional file 1: Figure S1. The same off-target effect was found in half of the offspring of mice carrying off-target locus. (A) Sanger Sequencing of the target PCR products (short band) of #1 off-target locus (Trp53 pseudogene) showed overlapping peaks in KI1 mouse but not in KI2 mouse. BLAST of the PCR products of #1 off-target locus (Trp53 pseudogene) showed tons of mismatches since the gRNA region in KI1 mouse but not in KI2 mouse, indicating the off-target effects of locus #1 in KI1 mouse. (B) Sanger Sequencing of the PCR products of #1 off-target locus (Trp53 pseudogene) showed overlapping peaks (indicating off-target effect) in half (4/8) of KI offspring of mice carrying #1 off-target locus. (C) BLAST of the PCR product of #1 off-target locus (Trp53 pseudogene) showed tons of mismatches since the gRNA region (indicating off-target effect) in half (4/8) of KI offspring of mice carrying #1 off-target locus. [file 12896_2019_573_MOESM1_ESM.tif]

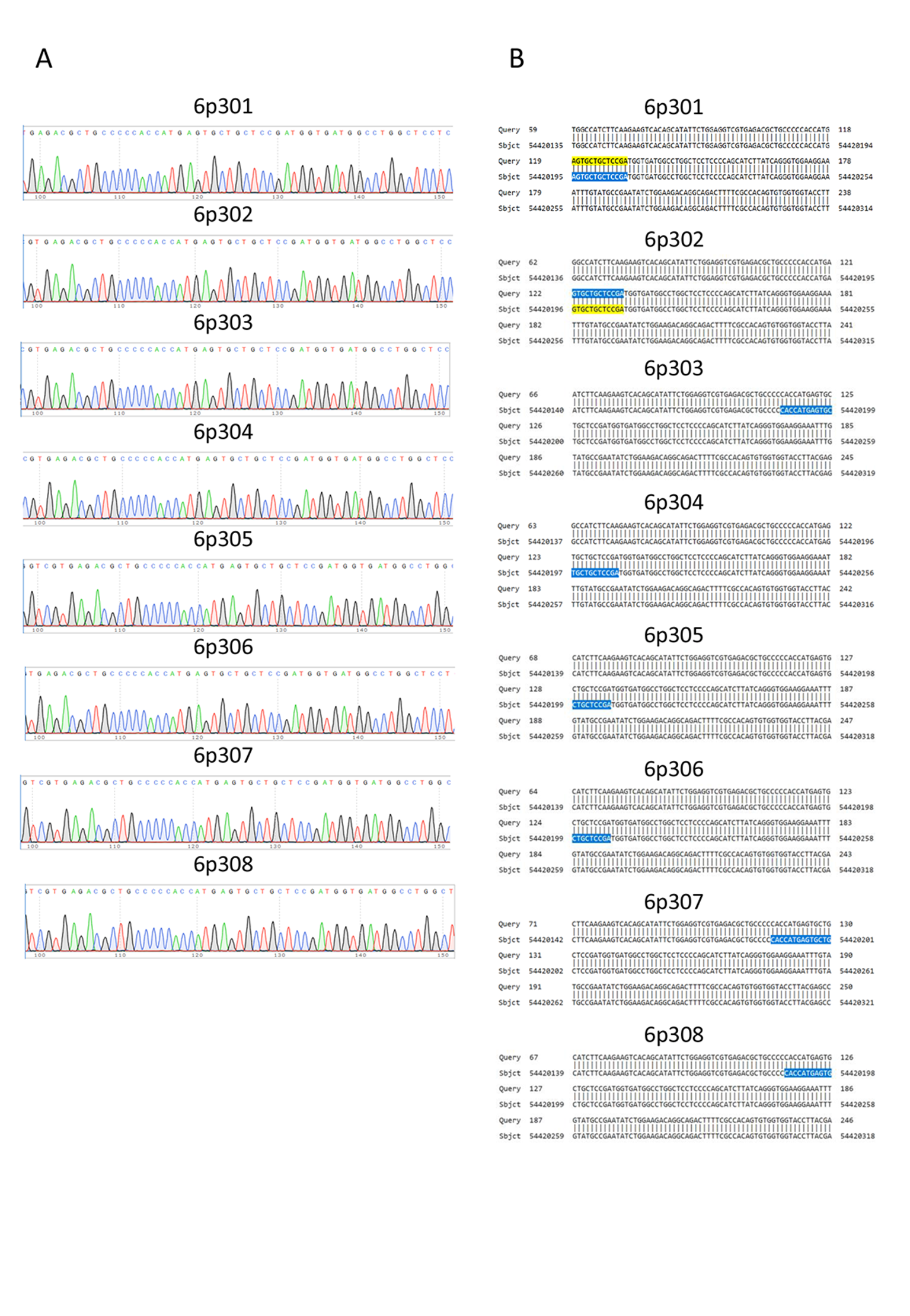

Supplement: Supplementary file 2 — Additional file 2: Figure S2. No off-target effects were found in all the offspring of mice that did not carry off-target locus. (A) PCR identifications of KI offspring of mice not carrying #1 off-target locus were validated by gel electrophoresis. (B) Sanger Sequencing of the PCR products of #1 off-target locus (Trp53 pseudogene) showed none of overlapping peaks (indicating off-target effect) in all of 8 offspring of mice without off-target effect. (C) BLAST of the PCR product of #1 off-target locus (Trp53 pseudogene) confirmed none of off-target effect in all of 8offspring of mice not carrying #1 off-target locus. [file 12896_2019_573_MOESM2_ESM.tif]

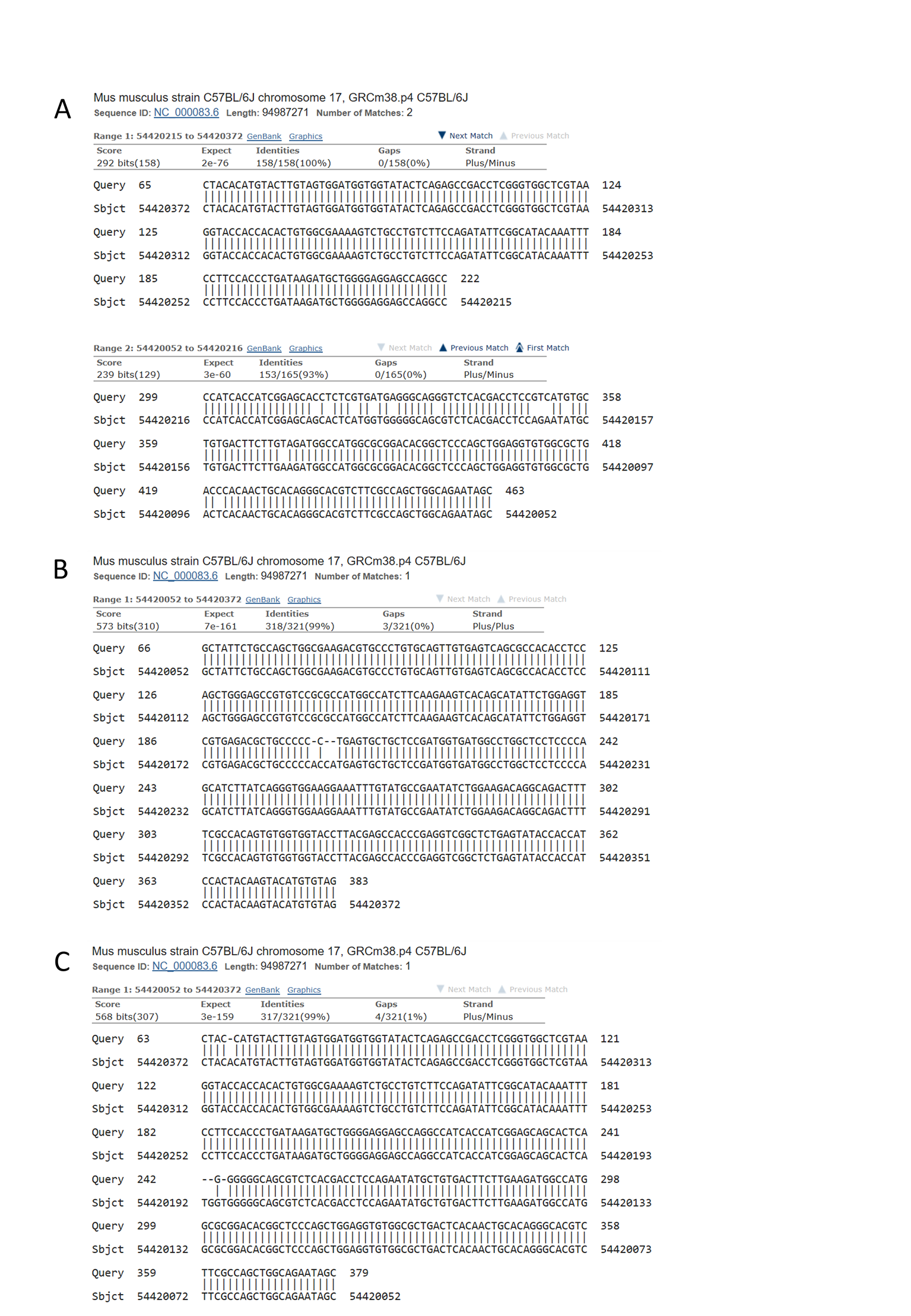

Supplement: Supplementary file 3 — Additional file 3: Figure S3. TA cloning and Sanger sequencing dissected the mutations of #1 off-target locus (Trp53 pseudogene). (A) TA clones of PCR products of #1 off-target locus were subjected to Sanger sequencing for analysing the detailed genomic mutations in #1 off-target locus. Sequence alignments showed that there were 75 bp insertion (222–299) in the #1 off-target locus. (B) Sequence alignments showed 3 bp deletion in the #1 off-target locus. (C) Sequence alignments of another clone showed 3 bp deletion in the #1 off-target locus. [file 12896_2019_573_MOESM3_ESM.tif]

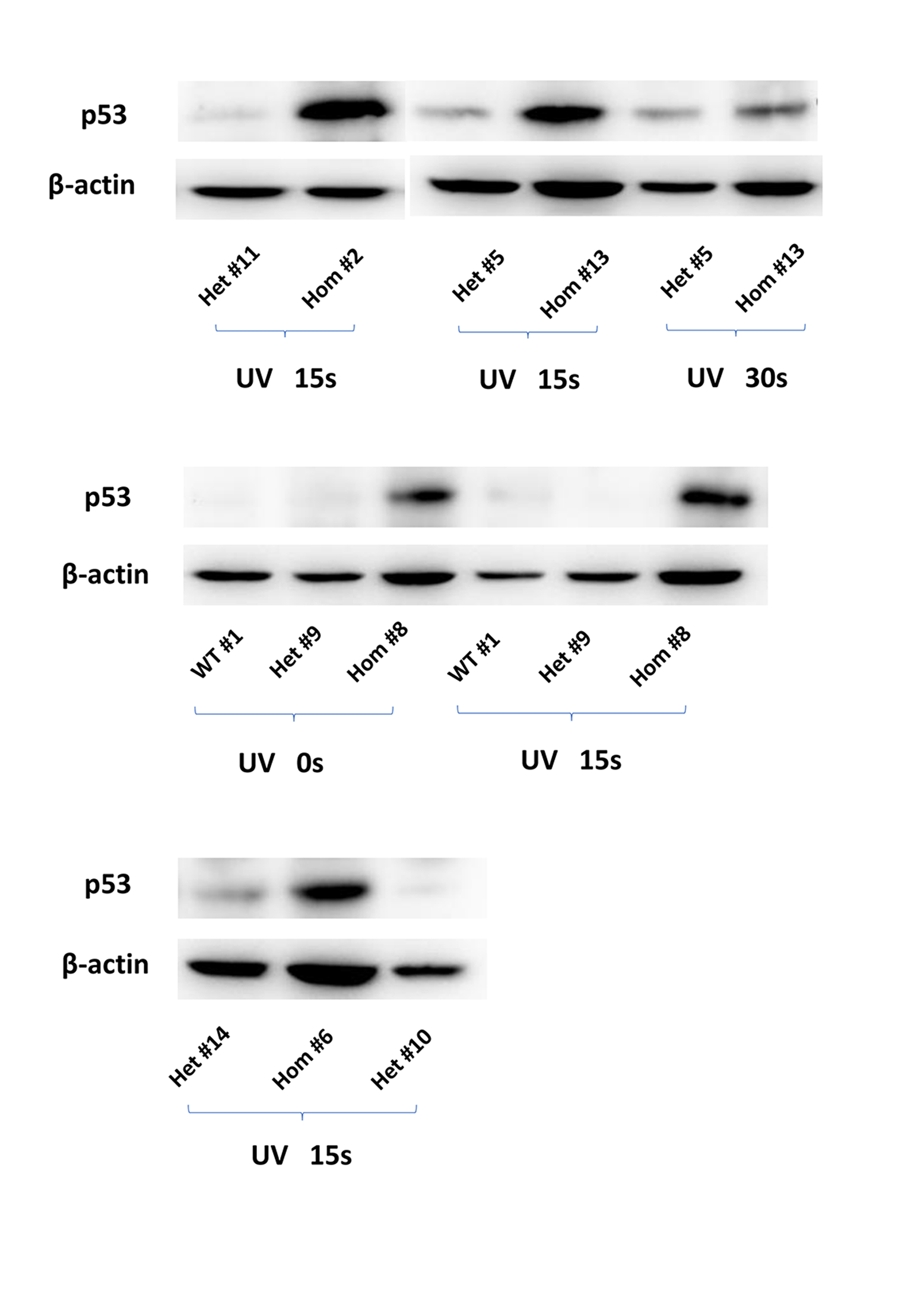

Supplement: Supplementary file 4 — Additional file 4: Figure S4. p53 level in the MEFs upon the stimulation of UV radiation. The protein levels of p53 in MEFs of various genotypes are compared upon UV stimulation of indicated time. The result showed that the expression of p53 increased in all Homozygous MEF cells. β-Actin worked as normalization control. [file 12896_2019_573_MOESM4_ESM.tif]
