# Supplementary material for: Rapid generation and selection of Cas9-engineering TRP53 R172P mice that do not have off-target effects
Source: BMC Biotechnol. 2019 Nov 8;19:74. doi: 10.1186/s12896-019-0573-z (PMC6839086; doi:10.1186/s12896-019-0573-z)
Supplement: Supplementary file 6 — Additional file 6: Data 1. oligos used in p53 R172P knockin. [file 12896_2019_573_MOESM6_ESM.pdf]

p53 R172P, exon5, G155C in mouse

gRNA1 N5R: tcggagcagcgctcatggtg

gRNA PCR primers:

p53 gRNA-F:

TAATACGACTCACTATAGGtcggagcagcgctcatggtgGTTTTAGAGCTAGA

AATAGC

gRNA-R: AAAAGCACCGACTCGGTGCC 125bp

Cas9 PCR primers:

Cas9-F:

TAATACGACTCACTATAGGGAGAATGGACTATAAGGACCACGAC

Cas9-R: GCGAGCTCTAGGAATTCTTAC about 4300bp

p53 donor: N120R, 6sense mutations

ggagatgggaggctgccagtcctaaccccacaggcggtgttgagggcttaccatcaccatcg

gagcaCcTctcGtgAtgAgggcagGgtctcacgacctccgtcatgtgctgtgacttct

genotyping primers:

p53 Mu-F: CCTTGACACCTGATCGTTACTC

p53 Mu-R: TCTCCCAGAGACTGCTGTTA 607bp
