# Supplementary material for: Rapid generation and selection of Cas9-engineering TRP53 R172P mice that do not have off-target effects
Source: BMC Biotechnol. 2019 Nov 8;19:74. doi: 10.1186/s12896-019-0573-z (PMC6839086; doi:10.1186/s12896-019-0573-z)
Supplement: Supplementary file 7 — Additional file 7: Data 2. The raw data collection. [file 12896_2019_573_MOESM7_ESM.pptx]

## Slide 1
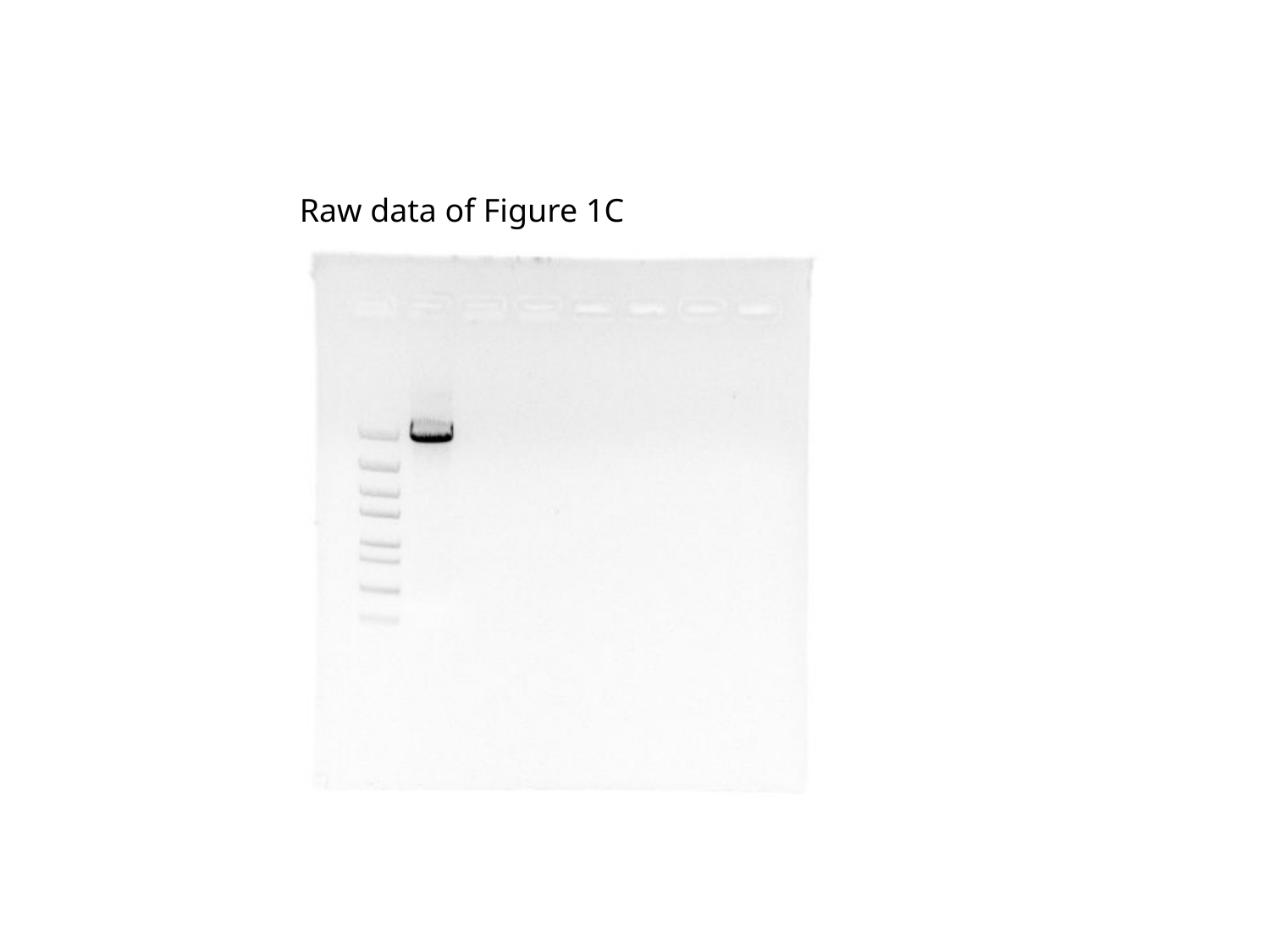

Raw data of Figure 1C

## Slide 2
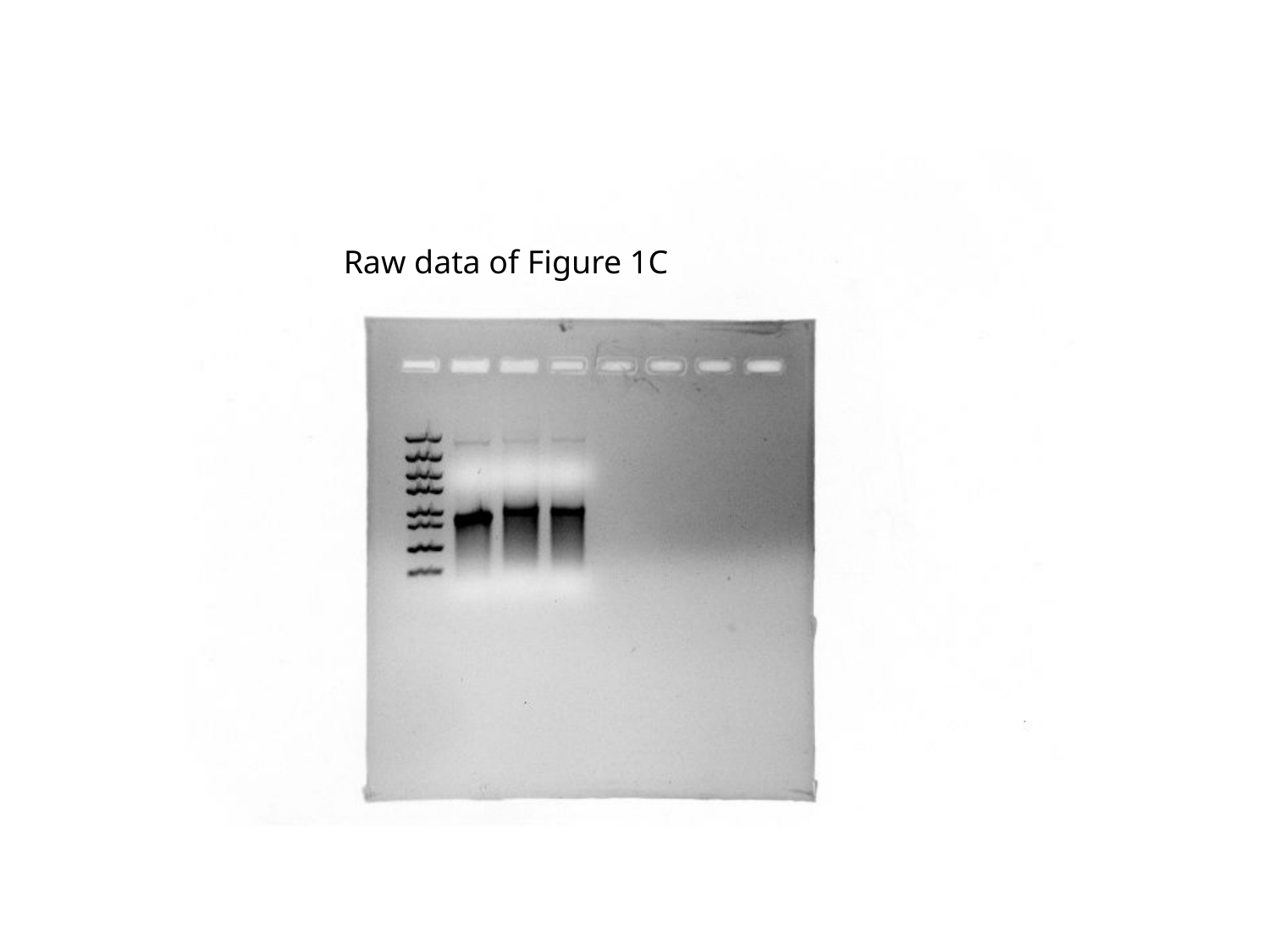

Raw data of Figure 1C

## Slide 3
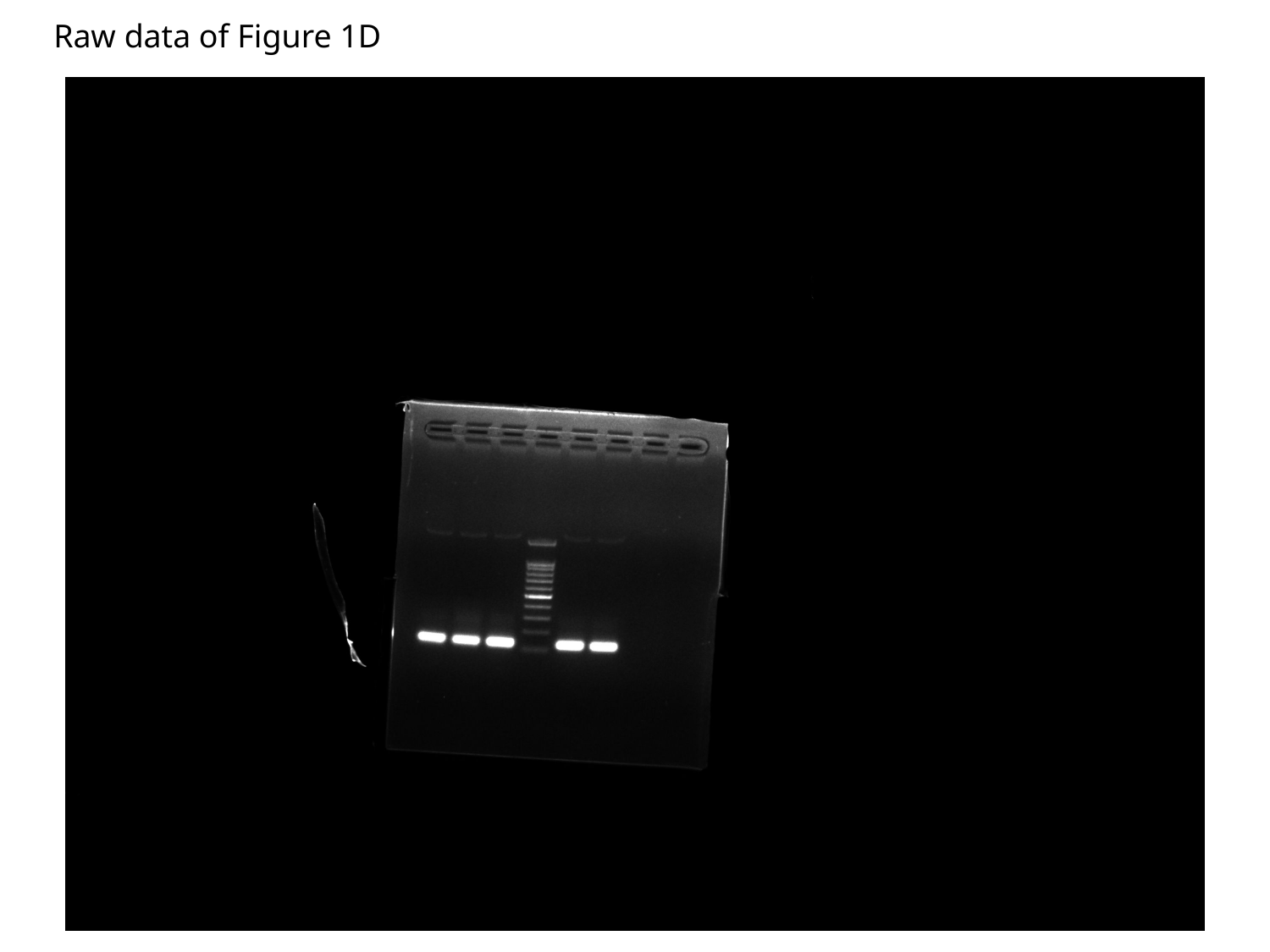

Raw data of Figure 1D

## Slide 4
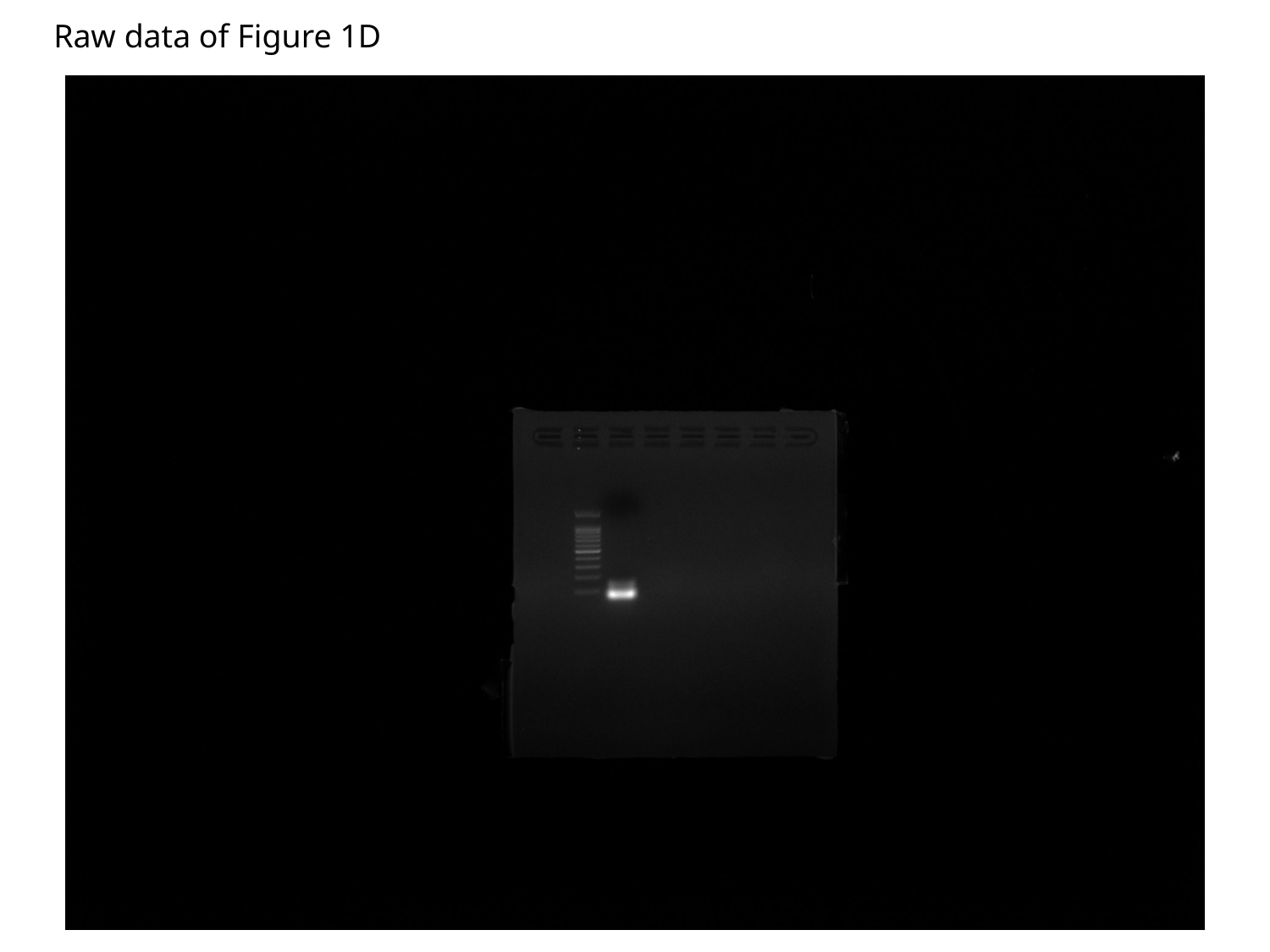

Raw data of Figure 1D

## Slide 5
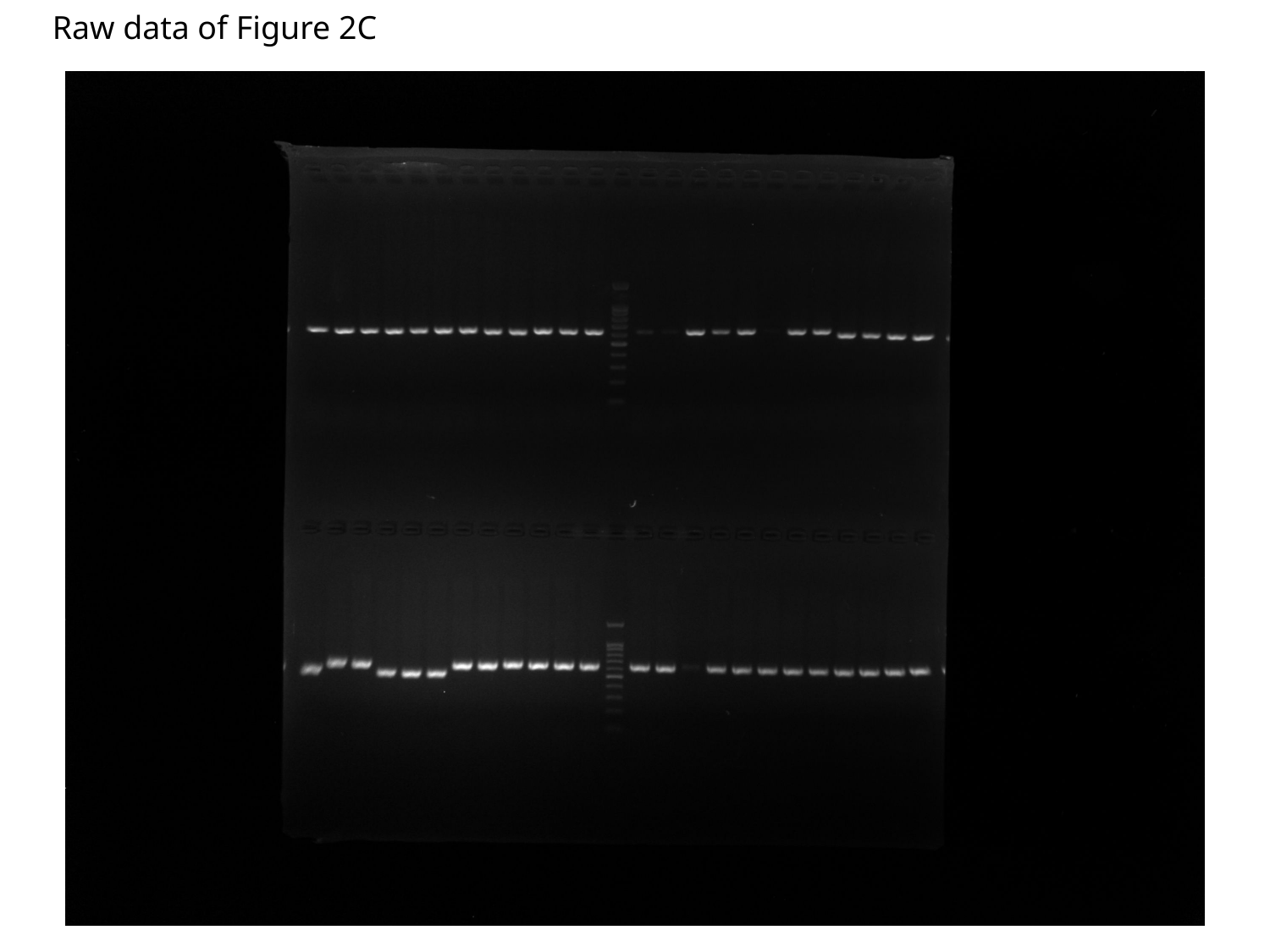

Raw data of Figure 2C

## Slide 6
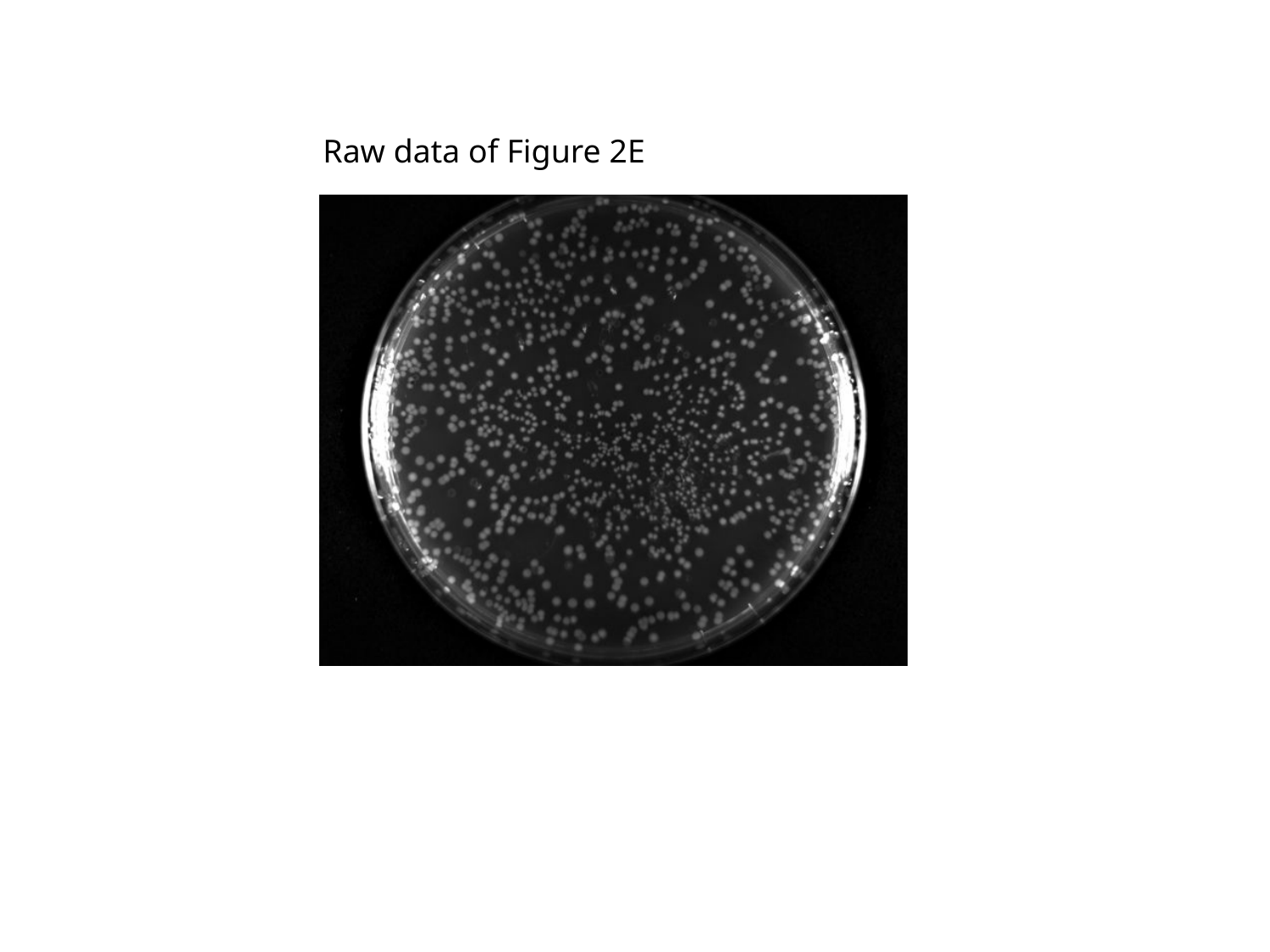

Raw data of Figure 2E

## Slide 7
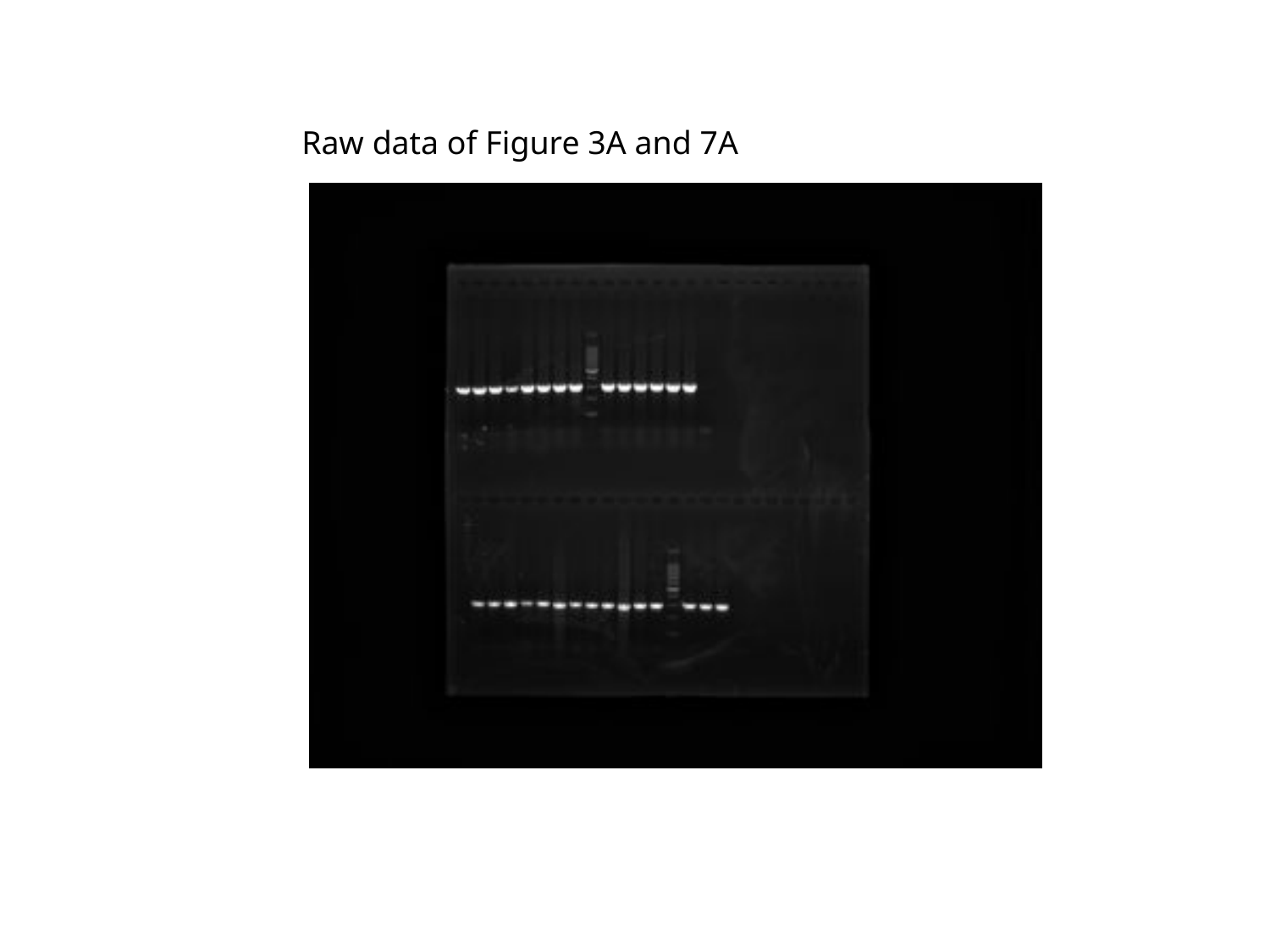

Raw data of Figure 3A and 7A

## Slide 8
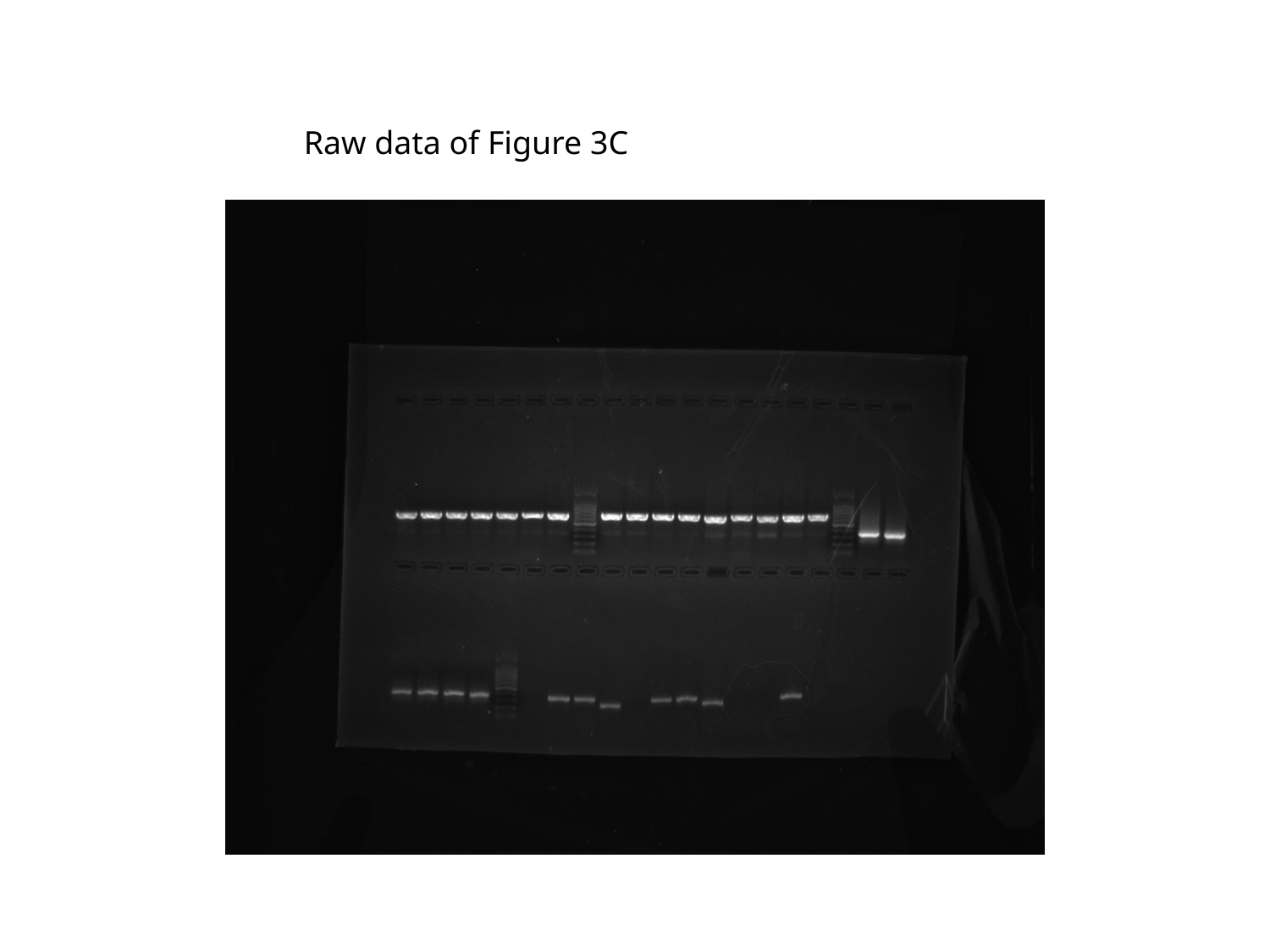

Raw data of Figure 3C

## Slide 9
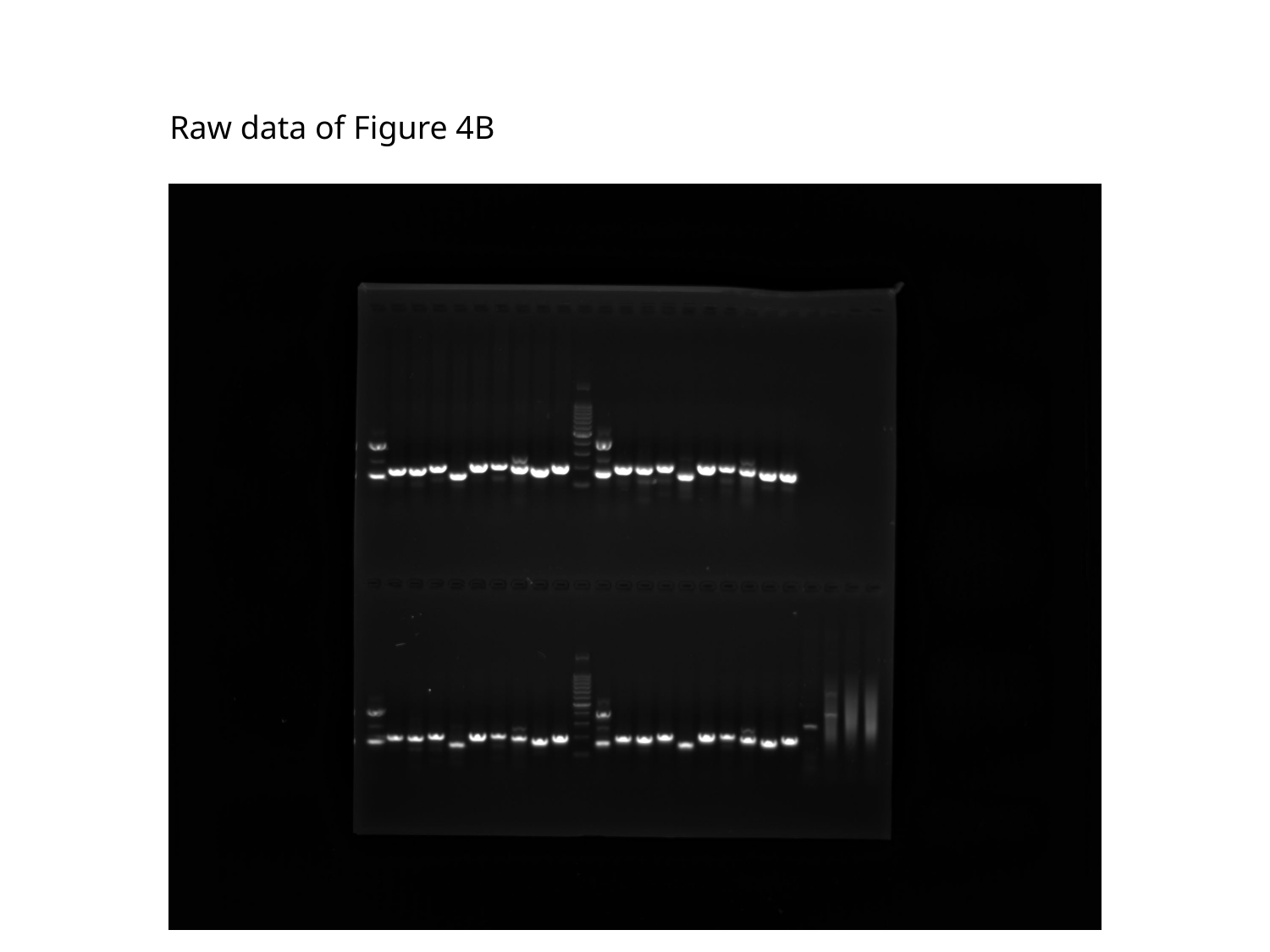

Raw data of Figure 4B

## Slide 10
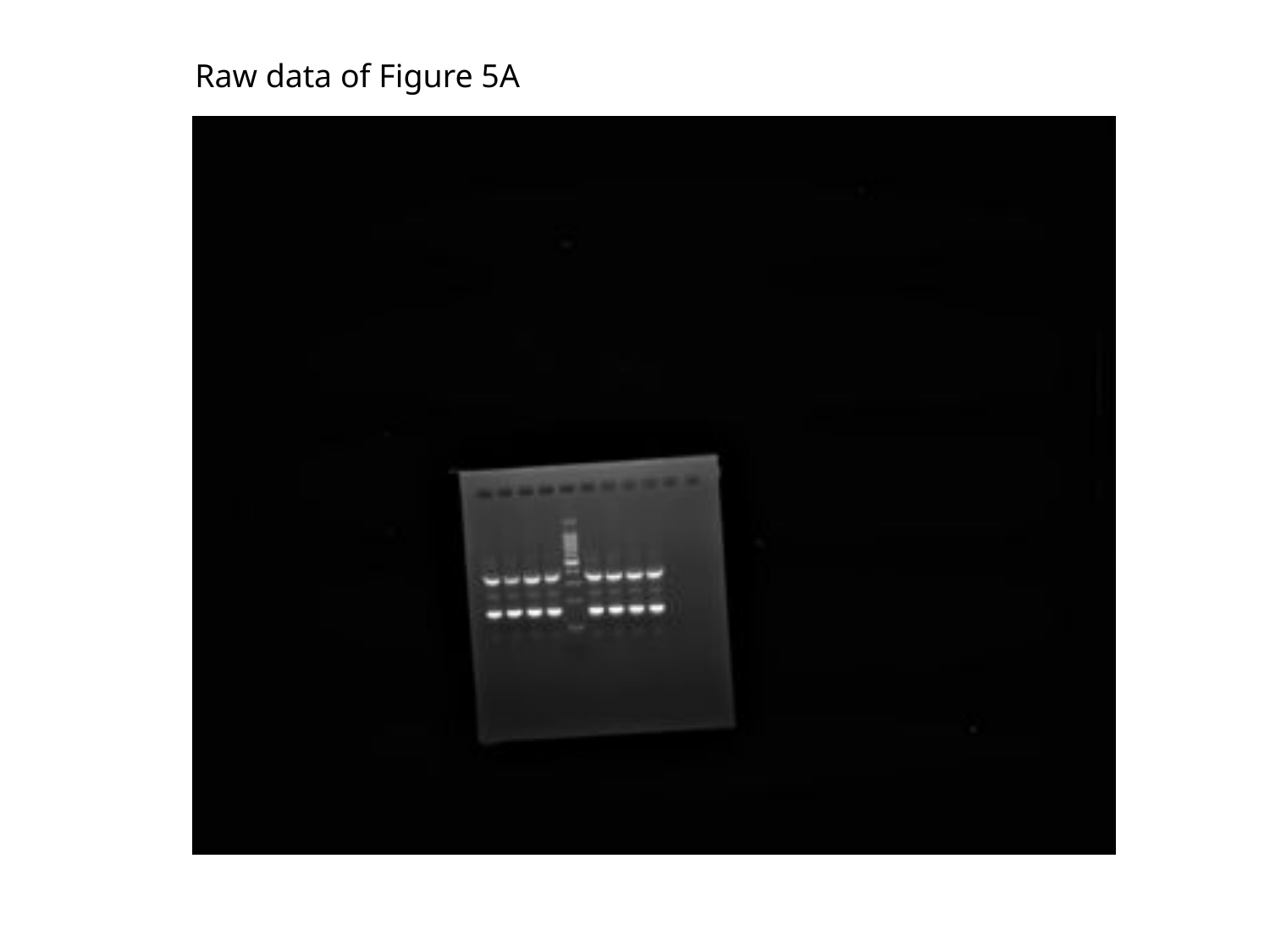

Raw data of Figure 5A

## Slide 11
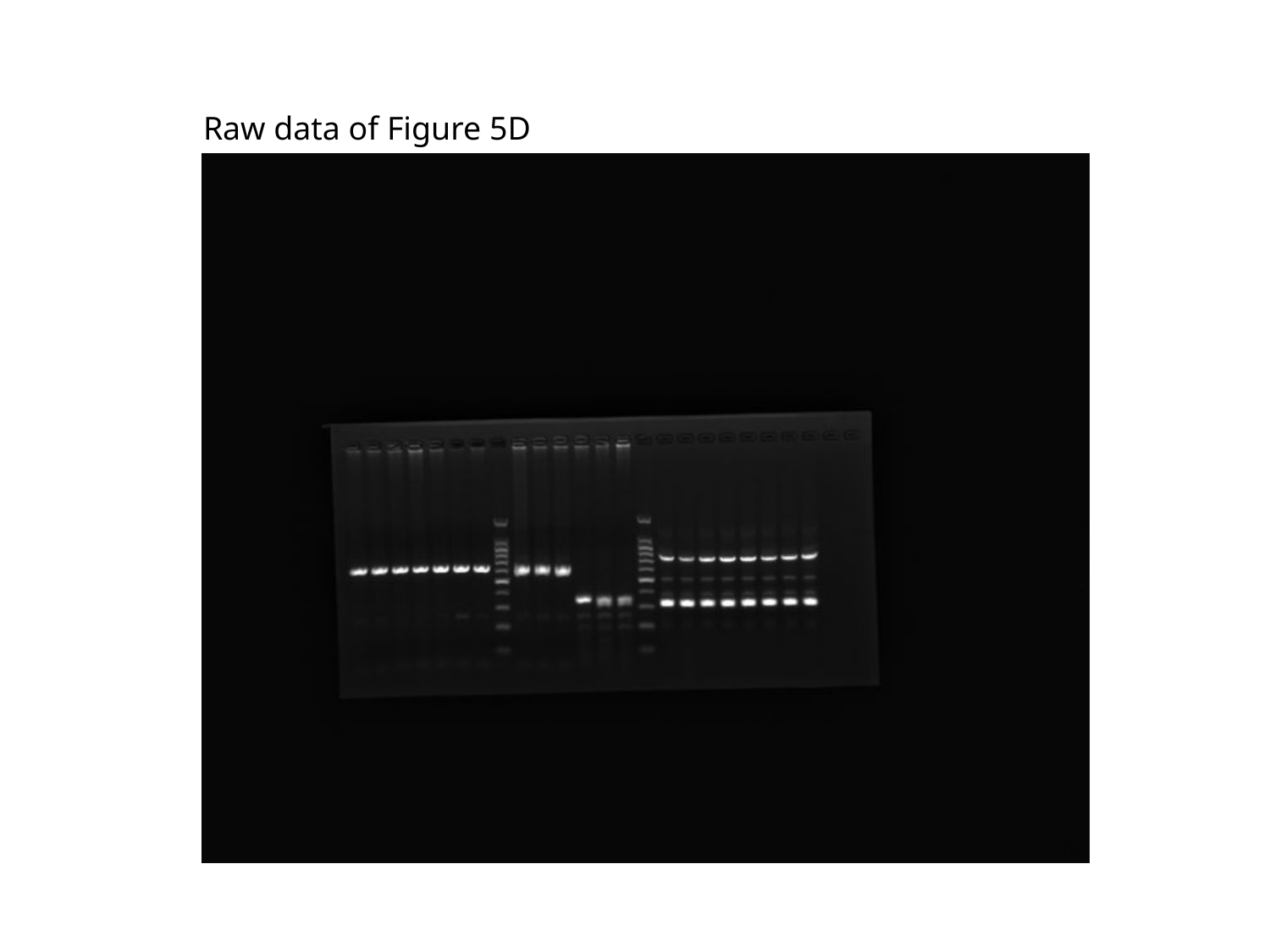

Raw data of Figure 5D

## Slide 12
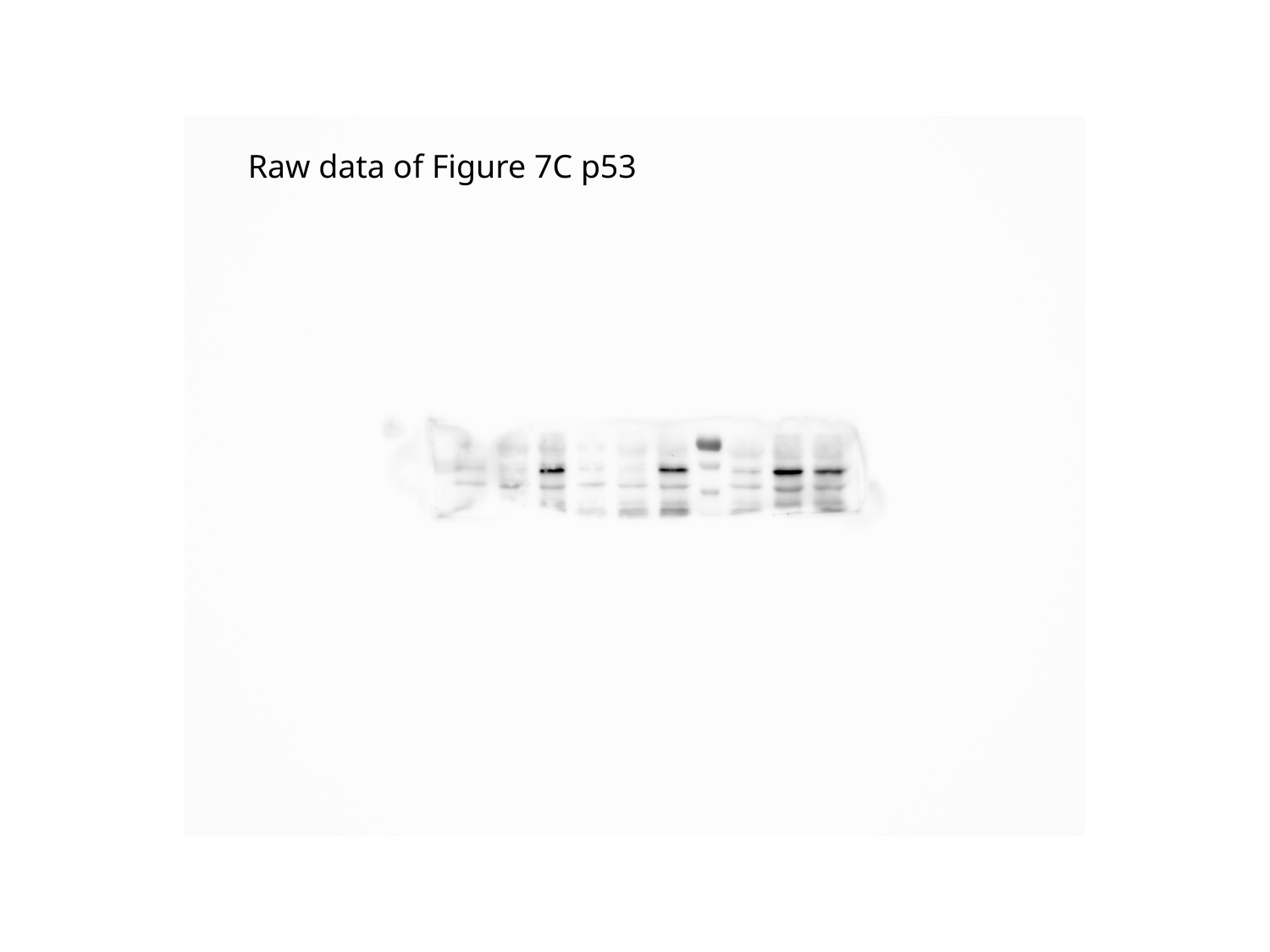

Raw data of Figure 7C p53

## Slide 13
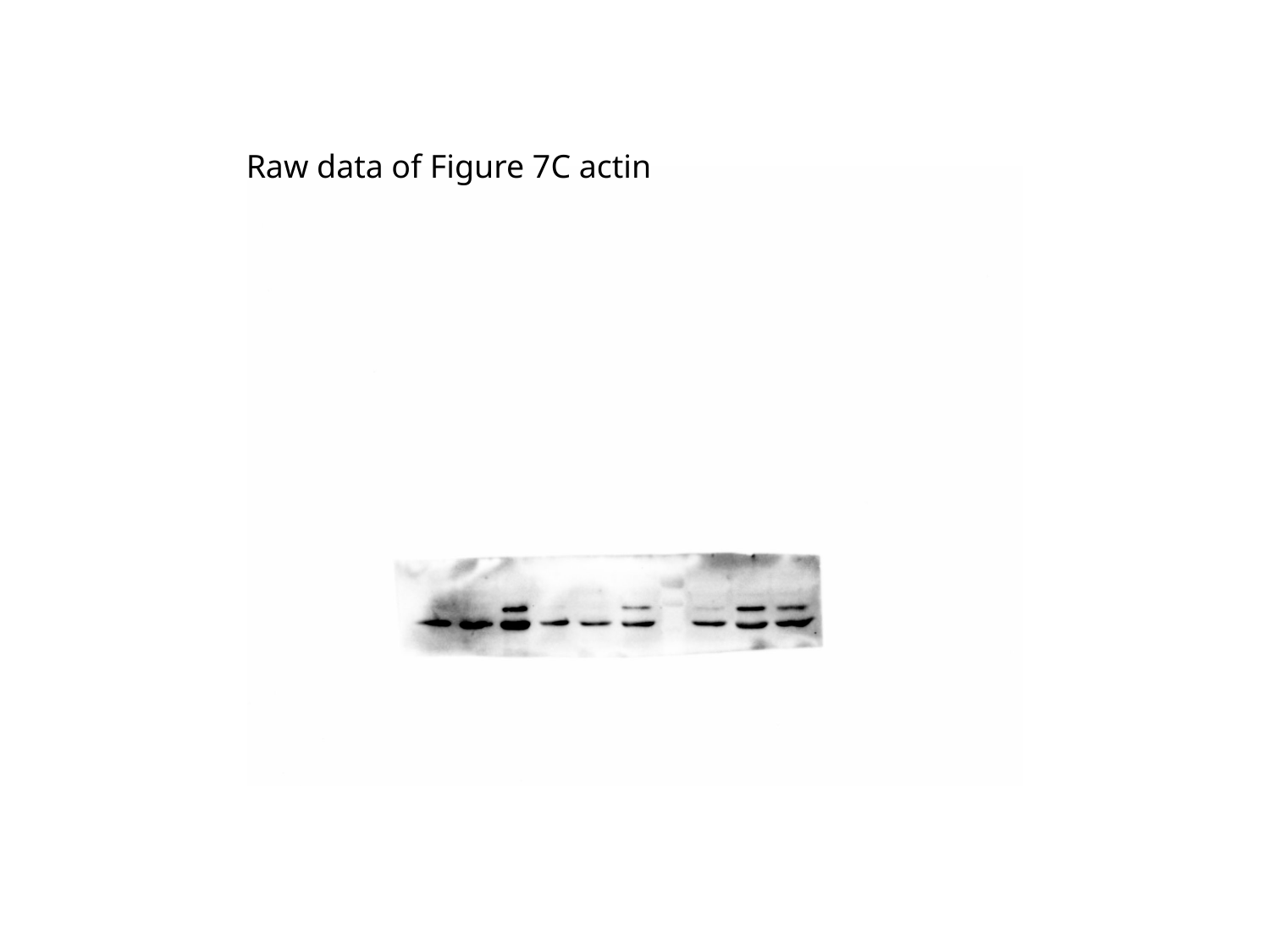

Raw data of Figure 7C actin

## Slide 14
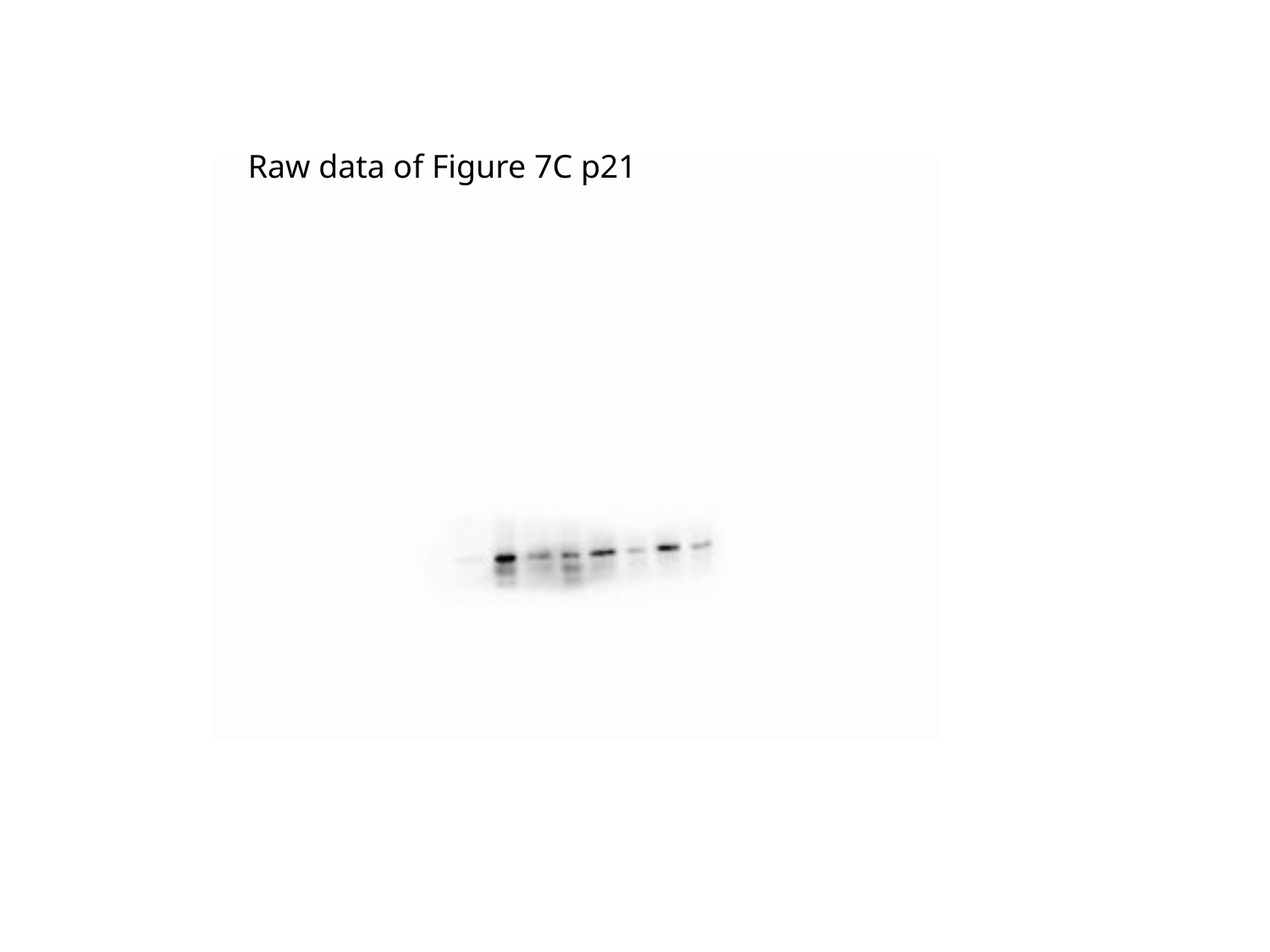

Raw data of Figure 7C p21

## Slide 15
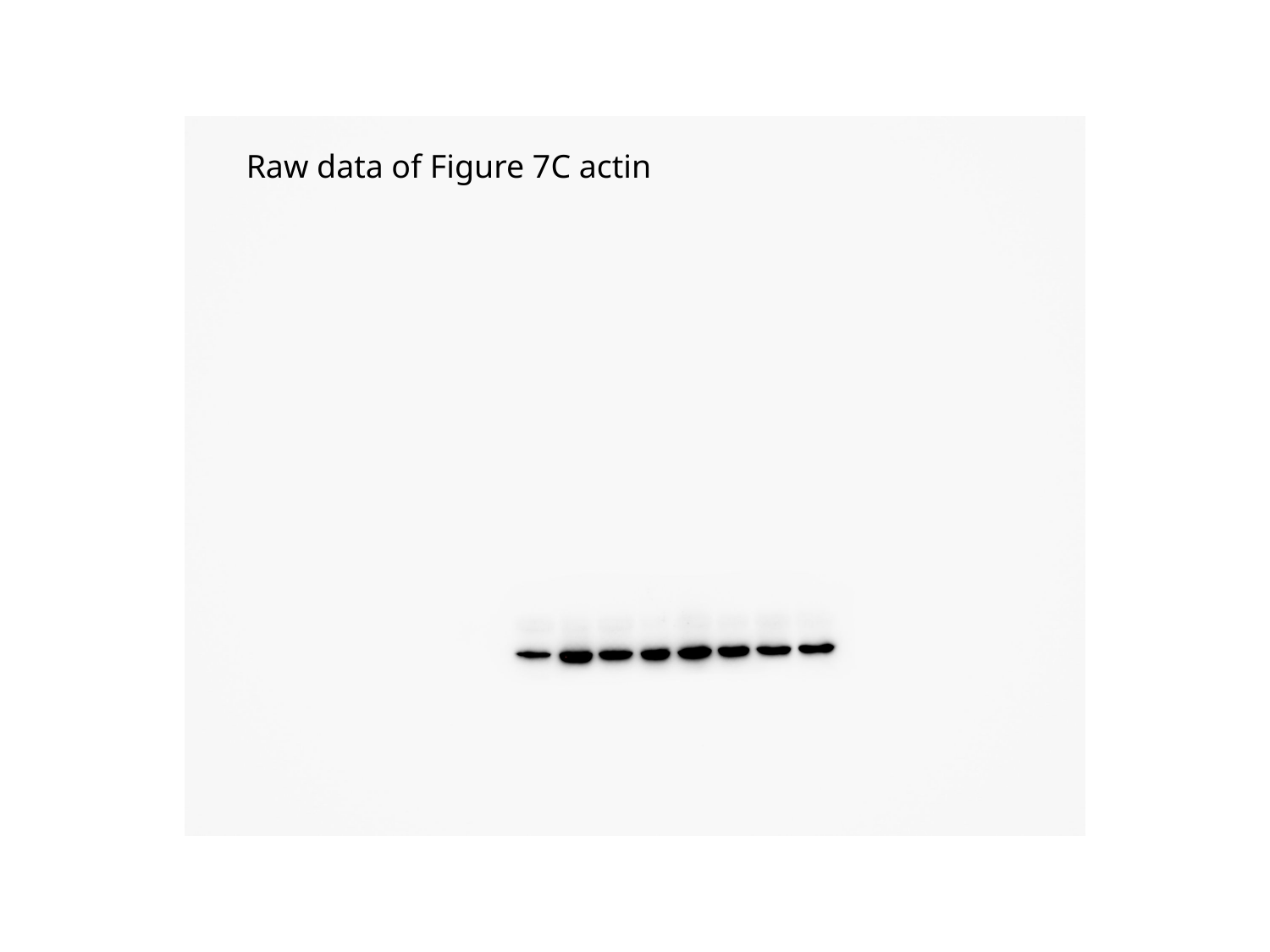

Raw data of Figure 7C actin

## Slide 16
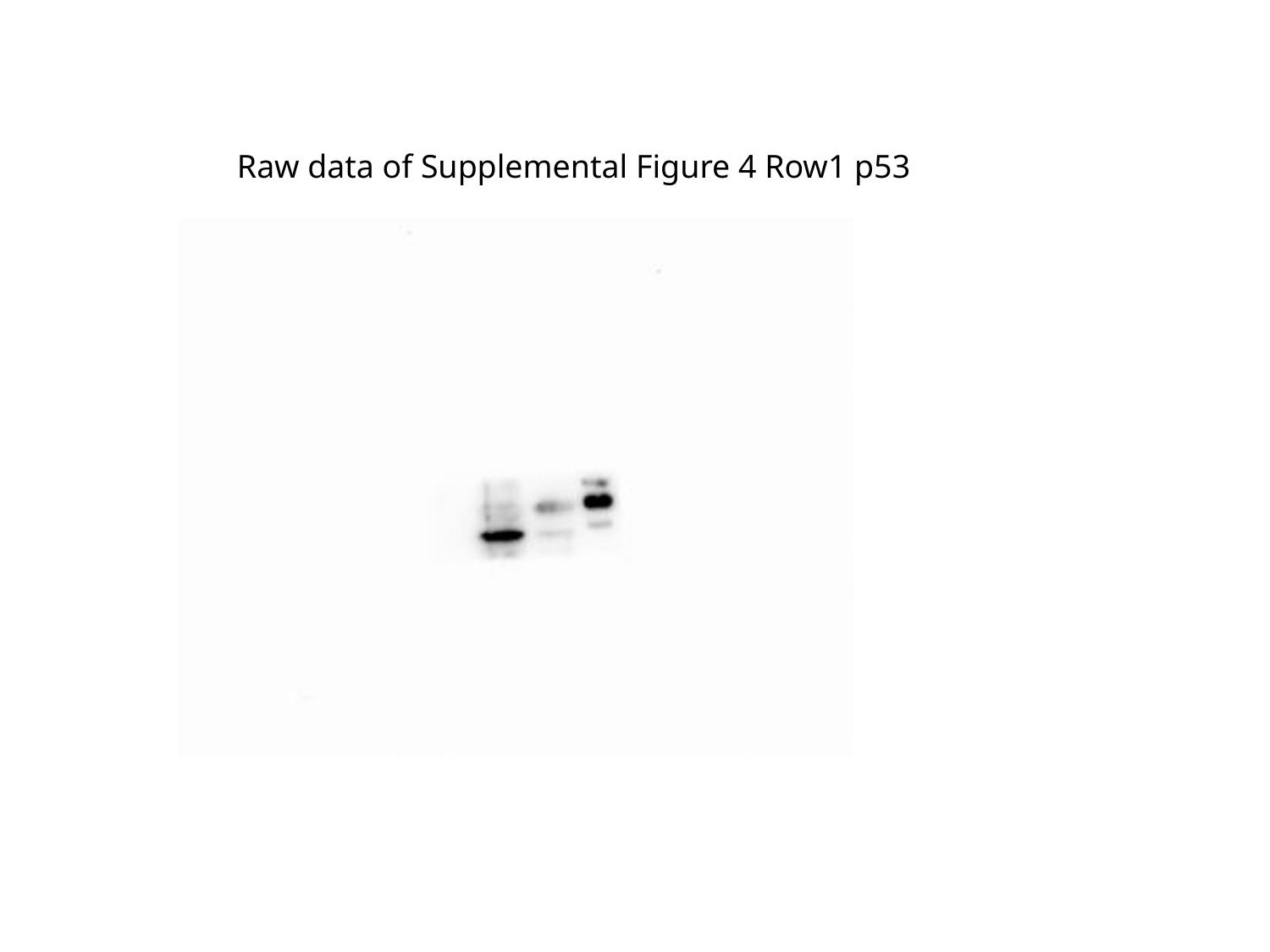

Raw data of Supplemental Figure 4 Row1 p53

## Slide 17
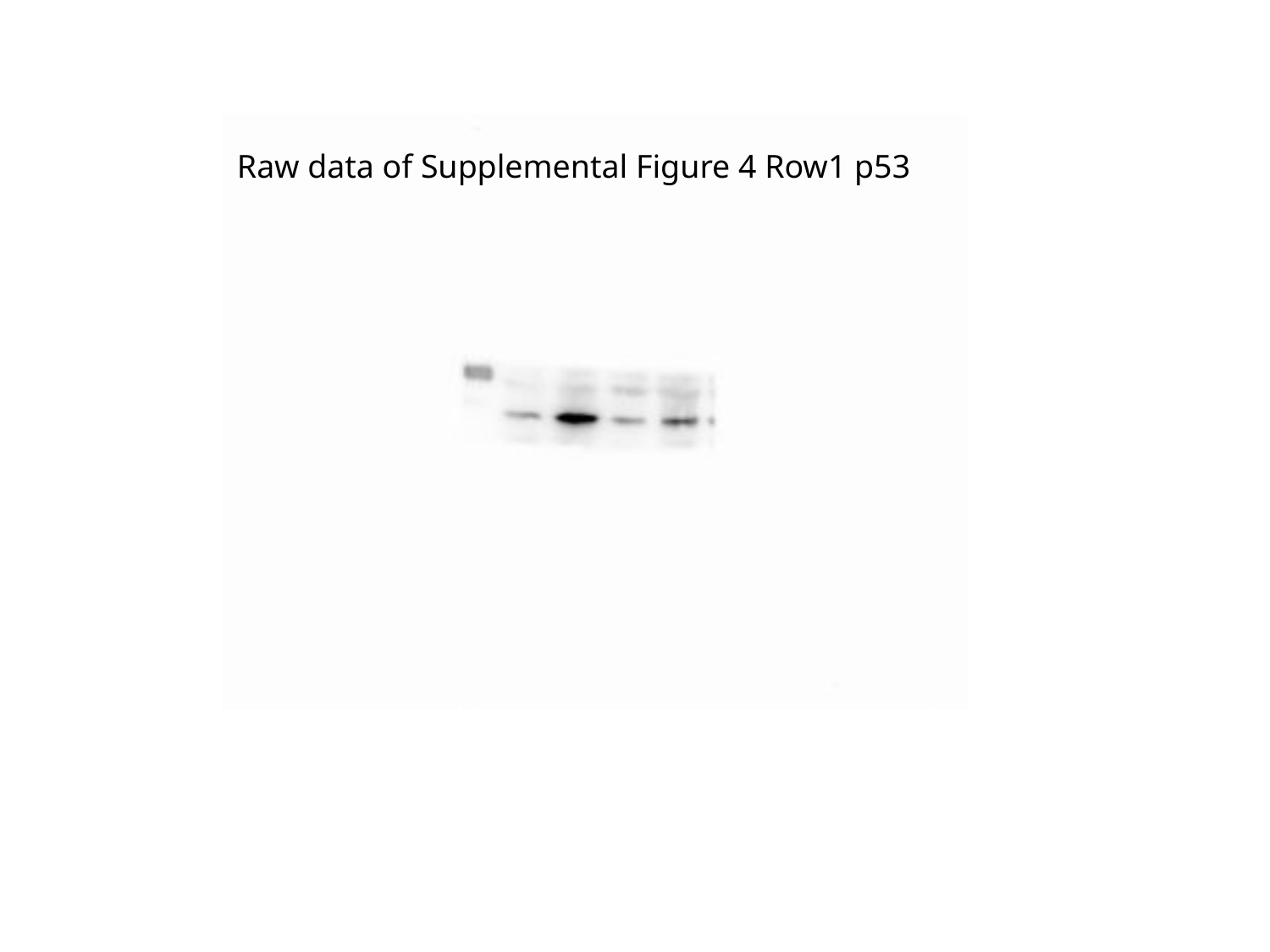

Raw data of Supplemental Figure 4 Row1 p53

## Slide 18
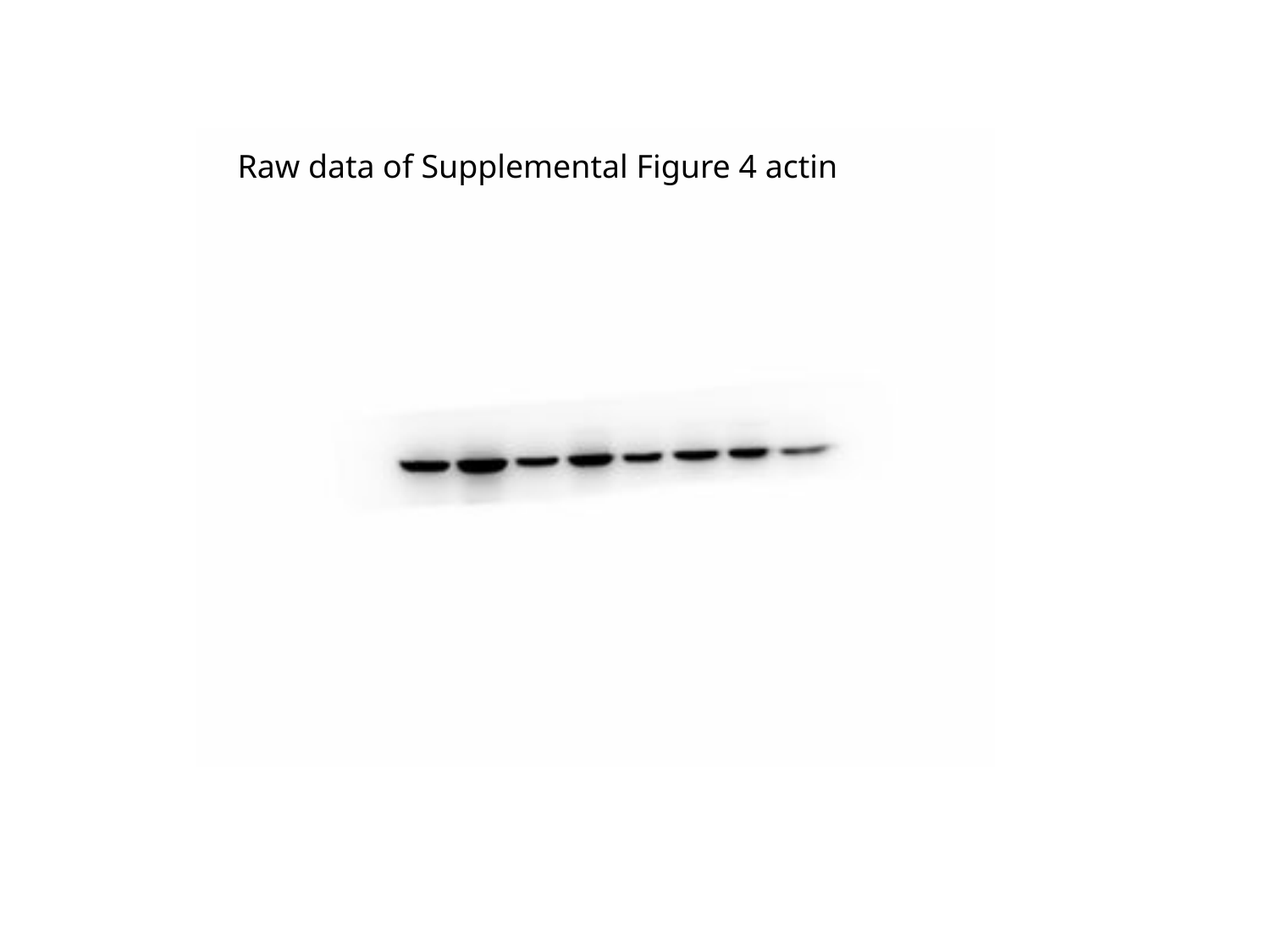

Raw data of Supplemental Figure 4 actin

## Slide 19
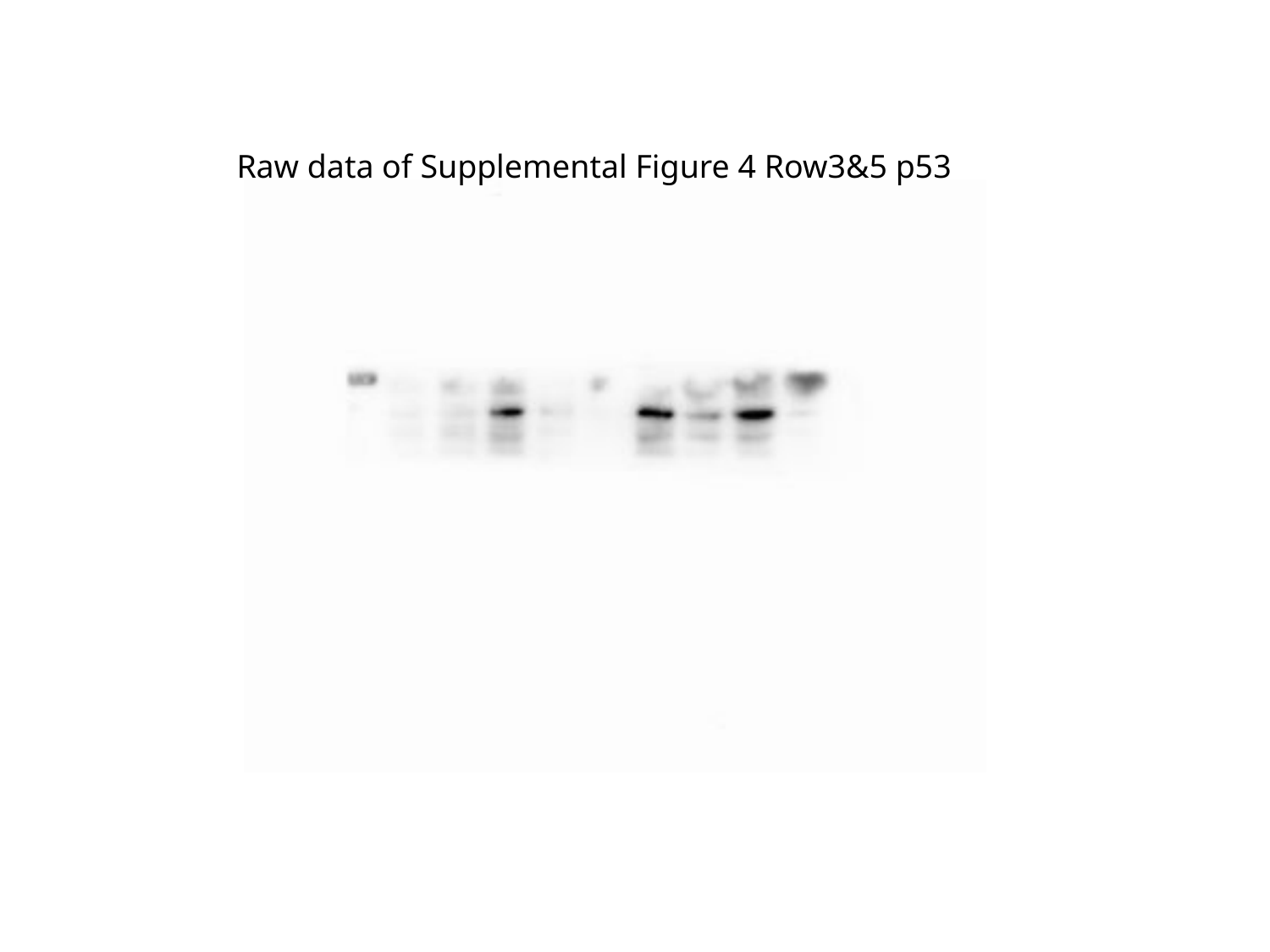

Raw data of Supplemental Figure 4 Row3&5 p53

## Slide 20
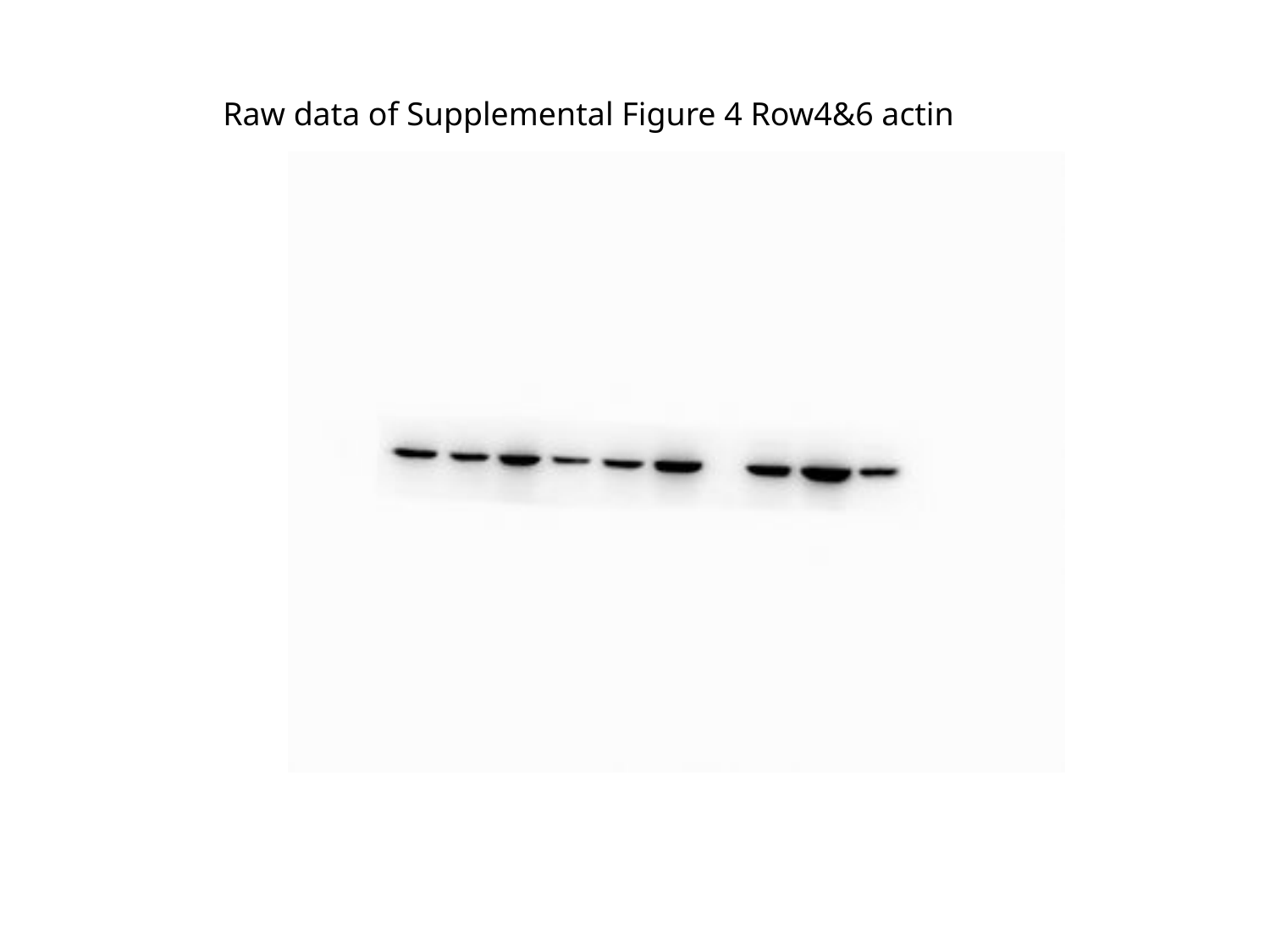

Raw data of Supplemental Figure 4 Row4&6 actin
